# Synthetic circuit-driven expression of heterologous enzymes for disease detection

**DOI:** 10.1021/acssynbio.1c00133

**Published:** 2021-08-31

**Authors:** Jiang He, Lior Nissim, Ava P. Soleimany, Adina Binder-Nissim, Heather E. Fleming, Timothy K. Lu, Sangeeta N. Bhatia

**Affiliations:** 1 Koch Institute for Integrative Cancer Research, Massachusetts Institute of Technology, Cambridge, MA, 02139, USA.; 2 Harvard–MIT Division of Health Sciences and Technology, Institute for Medical Engineering and Science, Massachusetts Institute of Technology, Cambridge, MA, 02139, USA.; 3 Synthetic Biology Group, Research Laboratory of Electronics, Department of Biological Engineering, Massachusetts Institute of Technology, Cambridge, MA, 02139, USA.; 4 Department of Biochemistry and Molecular Biology, The Institute for Medical Research Israel-Canada, Hadassah Medical School, The Hebrew University of Jerusalem, 91120, Jerusalem, Israel.; 5 Harvard Graduate Program in Biophysics, Harvard University, Boston, MA, 02115, USA.; 6 Department of Family Medicine, Meuhedet Health Maintenance Organization, Tel Aviv, Israel.; 7 Department of Electrical Engineering and Computer Science, Massachusetts Institute of Technology, Cambridge, MA, 02139, USA.; 8 Department of Medicine, Brigham and Women’s Hospital, Harvard Medical School, Boston, MA, 02115, USA.; 9 Broad Institute of Massachusetts Institute of Technology and Harvard, Cambridge, MA, 02139, USA.; 10 Wyss Institute at Harvard, Boston, MA, 02115, USA.; 11 Howard Hughes Medical Institute, Cambridge, MA, 02139, USA.

**Keywords:** biomarkers, proteases, activity probes, nanosensors, synthetic biology, nanotechnology, cancer

## Abstract

The integration of nanotechnology and synthetic biology could lay the framework for new classes of engineered biosensors that produce amplified readouts of disease states. As a proof-of-concept demonstration of this vision, here we present an engineered gene circuit that, in response to cancer-associated transcriptional deregulation, expresses heterologous enzyme biomarkers whose activity can be measured by nanoparticle sensors that generate amplified detection readouts. Specifically, we designed a AND-gate gene circuit that integrates the activity of two ovarian cancer-specific synthetic promoters to drive the expression of a heterologous protein output, secreted Tobacco Etch Virus (TEV) protease, exclusively from within tumor cells. Nanoparticle probes were engineered to carry a TEV-specific peptide substrate in order to measure the activity of the circuit-generated enzyme to yield amplified detection signals measurable in the urine or blood. We applied our integrated sense-and-respond system in a mouse model of disseminated ovarian cancer, where we demonstrated measurement of circuit-specific TEV protease activity both *in vivo* using exogenously administered nanoparticle sensors and *ex vivo* using quenched fluorescent probes. We envision that this work will lay the foundation for how synthetic biology and nanotechnology can be meaningfully integrated to achieve next-generation engineered biosensors.

## Graphical Abstract

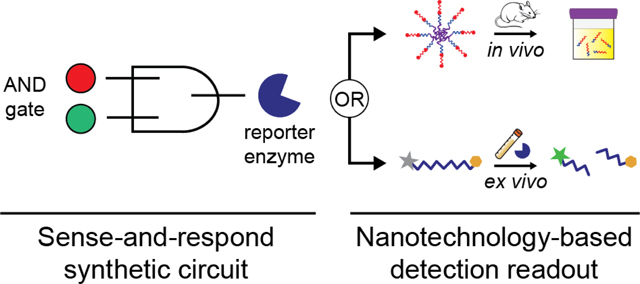

## Introduction

The computational principle of sense-and-respond is fundamental to engineering efforts that seek to build biological or chemical tools that can detect and report on the presence of disease. In synthetic biology, the forward engineering of biological systems towards this goal relies on the use of biological parts to create novel sense-and-respond functionalities inside living cells, enabling circuit-inspired control at the genetic, transcriptional, or protein levels^1–3^. In nanoparticle engineering, material and chemical systems are treated as the programmable entity and can be engineered as multifunctional, cooperative systems that can detect and respond to molecular events at the nanoscale^4,5^. In recent years, both these fields have seen the emergence of engineered, exogenous agents that can interrogate biological states *in vivo* to generate amplified readouts of disease^6^. Specifically, several technologies, both in synthetic biology and nanotechnology, have leveraged enzymatic activity to measure or produce biomarkers of disease^6–14^. Because they harness the unique substrate specificity and catalytic signal amplification properties of enzymes, these engineered activity-based diagnostics have the potential to overcome some of the sensitivity and specificity limitations of standard diagnostics. Within this sphere, the integration of synthetic biology with nanoparticle sensing tools could yield hybrid sensors that interface directly with the body to produce highly specific, amplified disease readouts.

Recent years have seen independent advances in the design of programmable diagnostics within each of synthetic biology and nanotechnology. Synthetic biology and genetic engineering tools have promoted the design and use of bacteriophages^15^, molecular recorders^13^, programmable probiotics^9,16^, genetically-encoded nanosensors^12^, and mammalian cells^10,17^ for disease detection applications. For example, probiotic bacteria have been programmed to serve as delivery vectors that conditionally produce secreted enzymes to generate a non-invasive, signal amplified readout in urine^9^. However, this system lacked any disease-specific sensing circuitry upstream of the activity-based enzymatic readout. To this end, molecular engineering tools have been leveraged to develop DNA vectors carrying a tumor-specific promoter driving expression of a reporter enzyme^13^. More recently, macrophages were engineered to secrete a heterologous reporter enzyme in response to adopting a tumor-associated (M2 macrophage) transcriptional profile, and were administered in mice with colorectal cancer to generate a sensitive, signal amplified disease readout that could be queried *ex vivo* in the blood^10^. In addition, gene therapy delivery techniques, such as engineered lentiviral vectors, have recently been tested both pre-clinically and clinically for *in vivo* therapeutic applications in cancer^18,19^ and other diseases^20,21^, and could enable the targeted delivery of synthetic disease-sensing gene circuits *in vivo*.

In parallel to these synthetic biology and gene therapy advances, nanoparticle sensors and chemical probes have been deployed to actively query disease activity *in vivo* to generate diagnostic readouts. Several such approaches convert the activity of enzymes, such as proteases, involved in disease progression into an imaging or biofluid-based detection readout^6,7,22^. For example, activity-based nanosensors, an emerging biosensor class, sense dysregulated protease activity at the site of disease and respond by releasing a renal clearable synthetic reporter that enables detection of disease via a simple urine test^11,16,23–25^. However, this and related approaches rely on endogenous protease dysregulation as the measured disease biomarker. Because proteases are expressed and active in healthy cells as well as in a variety of disease etiologies, this places a heavy engineering requirement to try to ensure sensor specificity.

When integrated with responsive nanoparticles or molecular probes, synthetic biology tools could open new diagnostic pathways by expanding the set of accessible input signals specific to a disease of interest, such as cancer. Namely, due to the variety of molecular parts available, such as synthetic promoters with enhanced cell-state specificity (SPECS)^26^, synthetic circuits based on transcriptional control are highly tunable and can be engineered to detect the altered transcriptional state present in cancer cells^1,2,27–29^. In turn, the sensed transcriptional signal could be transduced into an easily detectable, biorthogonal output, such as a heterologous reporter enzyme, which could then trigger the activation of a nanoparticle sensor to ultimately produce a highly specific, amplified readout. More generally, despite the foundational principles common to both synthetic biology and nanotechnology-inspired biosensors, they have yet to be meaningfully integrated. Simultaneously harnessing the programmability of genetically-controlled synthetic biology systems and the modular targeting, sensing, and reporting capabilities of nanomaterials could open new avenues in the design of engineered diagnostics and therapeutics for cancer and other diseases.

In this work, we explored how synthetic biology and nanoparticle sensors can be integrated to achieve a cooperative sense-and-respond system that produces amplified readouts of disease states. We engineered a synthetic gene circuit that, when integrated into cancer cells following lentiviral delivery, drives specific production of a heterologous enzyme biomarker by the modified tumor cells. The secreted heterologous enzyme turn catalyzes highly specific cleavage reactions measurable by nanoparticle sensors, enabling noninvasive, amplified readouts in either the blood or urine. As a proof of concept, we deployed this system in a mouse model of disseminated ovarian cancer, a disease with a dismal mortality rate in which endogenous, blood-based biomarkers face significant sensitivity limitations^30,31^. Our integrated system consisted of an initial synthetic biology-driven sensing phase followed by a nanotechnology-enabled detection phase. For the sensing phase, we engineered an RNA-based AND gate circuit that integrated the activity of two ovarian cancer SPECS^29^ via a Boolean AND gate and generated a biomarker output only when both promoters were simultaneously active. The circuit was delivered into ovarian tumors, where it could sense aberrant cancer-associated transcriptional activity and drive the tumor-specific expression of a heterologous biomarker, the Tobacco Etch Virus (TEV) protease^32,33^. Because of its stringent cleavage preference and orthogonality to endogenous proteases in the body, TEV is particularly well suited for use as a heterologous biomarker. For the detection phase, nanoparticle probes carrying the cognate peptide substrate for TEV were used to measure the activity of this enzyme *in vivo* or *ex vivo* to generate amplified signals detectable in the urine or blood, respectively. This work presents a new paradigm for the integration of synthetic gene circuits and nanotechnology towards the development of engineered sense-and-respond diagnostic or therapeutic systems.

## Results

### Sensing ovarian cancer-associated transcriptional dysregulation with an engineered synthetic circuit

**Figure 1.**
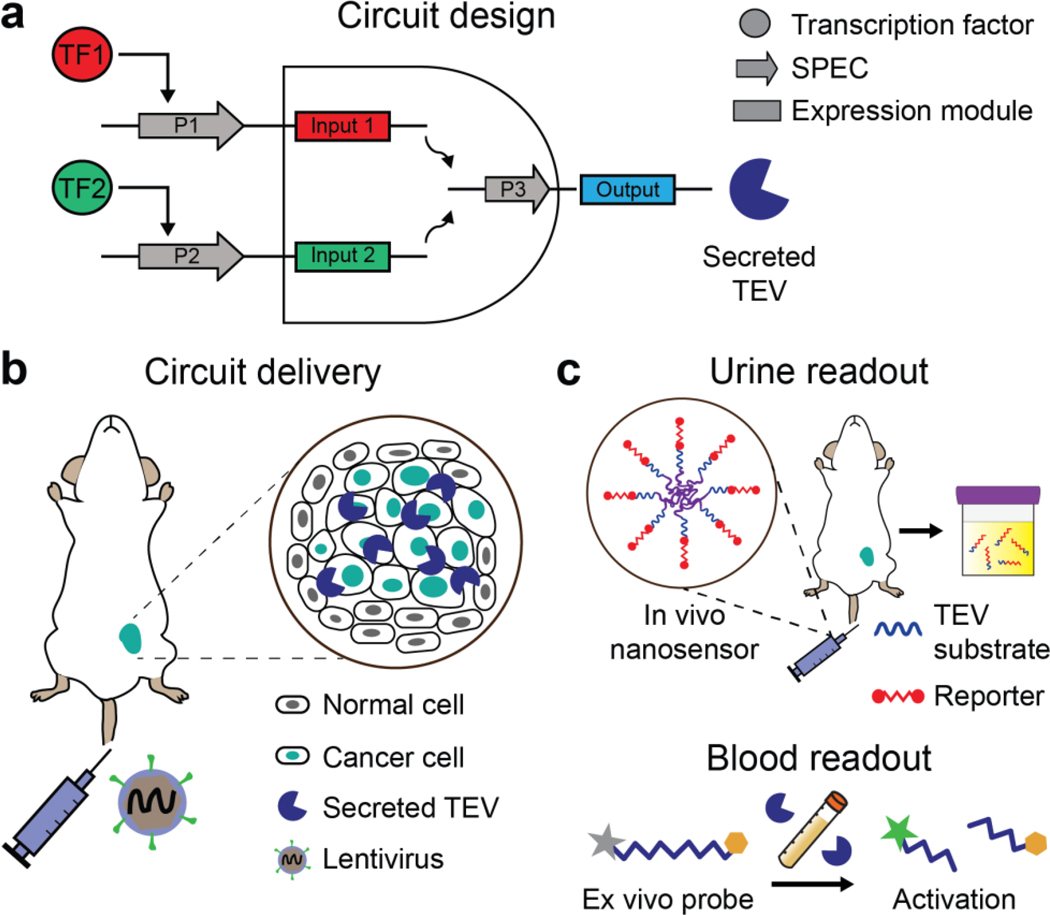
Overview of the activity-based sense-and-respond system. **A)** To detect or monitor the presence of cancer cells, an RNA-based AND gate is used to express a heterologous biomarker (secreted TEV protease) that can be detected either in the urine or blood. In response to each of two cancer-associated transcription factors (TF1 and TF2), cognate SPECS (P1 and P2) drive the expression of input module 1 or 2, respectively (Input 1 and Input 2). Only when both input modules are triggered will the heterologous biomarker be expressed and secreted. **B)** To implement the synthetic circuit *in vivo*, three viruses, each bearing one of the input (1 or 2) or output gene modules, are intraperitoneally (i.p.) injected into tumor-bearing mice. Successful transduction leads to subsequent expression of the AND-gated heterologous reporter enzyme in cancer cells, in response to the cancer-associated transcription factors that provide the upstream circuit inputs. **C)** The sense-and-respond system can yield an activity-based signal to be read out either in the urine or in the blood. For the urine readout (top), mice were intravenously (i.v.) administered an *in vivo* nanosensor consisting of a reporter-tagged, TEV-specific peptide substrate conjugated to the surface of a poly(ethylene glycol) (PEG) carrier. When exposed to active TEV protease *in vivo*, reporter peptides are liberated from the TEV-sensitive nanosensor and accumulate into the urine where they are measured via immunoassay. For the blood readout (bottom), a quenched fluorescent reporter was designed to measure, *ex vivo*, the activity of TEV protease present in plasma. Cleavage by TEV proteases results in fluorescence dequenching and signal generation.

Our integrated sense-and-respond platform consists of (1) a synthetic circuit that drives tumor-specific expression of the heterologous biomarker TEV protease during the sensing step, and (2) two readout methods to measure the subsequent protease activity during the detection step (Fig. 1). The input component of the circuit integrates the activity of two ovarian cancer SPCES^26,29^ (P1 and P2) to sense cancer-associated transcriptional activity in circuit-expressing cells and to ultimately produce secreted TEV protease (Fig. 1A). To implement the synthetic circuit *in vivo*, we used three separate lentiviruses that encode the input modules (*Module 1* and *Module 2*) and the output module (*Module* 3) to transduce ovarian cancer cells in tumor-bearing mice (Fig. 1B). Sensing and transduction of the transcription factor input signals resulted in expression and secretion of TEV protease (Fig. 1A). In turn, the activity of secreted TEV protease was subsequently queried either by responsive nanoparticles *in vivo* or by a Förster resonance energy transfer (FRET)-based detection assay *ex vivo*, to ultimately generate an amplified readout in either the urine or blood, respectively (Fig. 1C).

**Figure 2.**
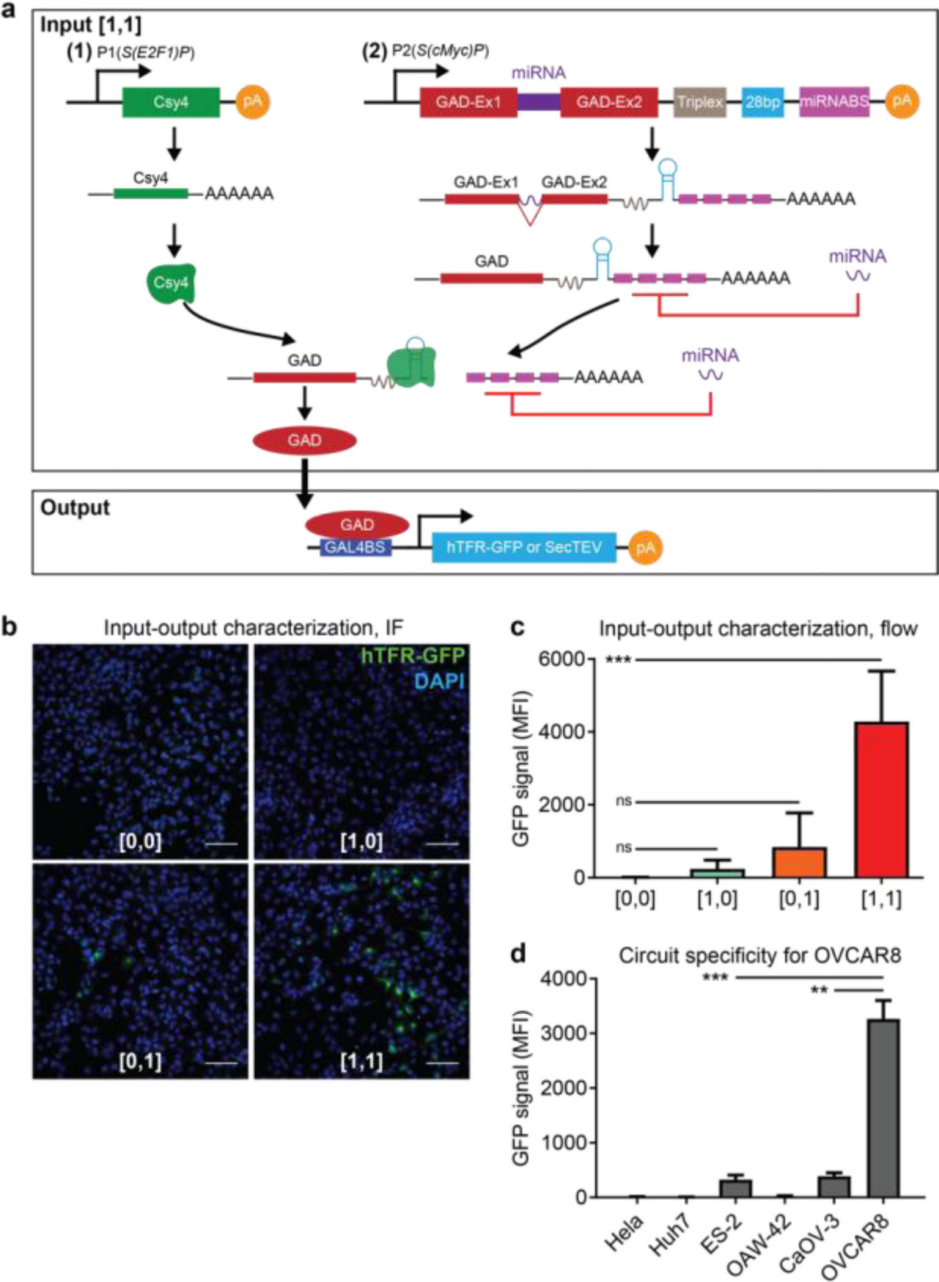
Design and validation of the synthetic sense-and-respond circuit. **A)** Input 1 relies on a SPEC (S(*E2F1*)P) to drive the specific expression of endoribonuclease Cys4. Input 2 relies on a SPEC (S(*cMyc*)P) to drive the transcription of a fusion protein, GAD, consisting of the GAL4 DNA binding domain and the transcriptional transactivator VP16, and a miRNA transcript. The 3’ end of GAD transcripts has a hairpin structure for Csy4 binding and a miRNA binding site. When both inputs are present ([1,1] state), Csy4 will bind to the hairpin structure to release the inhibitory effect of the miRNA on GAD translation, driving the expression of GAD. The output expression is driven by a synthetic promoter GAL4BS, which is targeted by transcription activator-binding domain fusion, GAD. Two outputs are used in this study: membrane GFP (hTFR-GFP) or secreted TEV protease (SecTEV). **B)** OVCAR8 cells were either transduced with the output virus carrying a membrane GFP expression cassette ([0,0]), or the output virus with one of two input viruses ([1,0], [0,1]), or both input viruses and the output virus ([1,1]). After two days, cells were fixed for immunofluorescence imaging or flow cytometry. Scale bar: 100μm. **C)** Quantification of flow cytometry analysis of mean GFP fluorescent intensity with different input and output configurations. Mean ± s.d.; N=3; ordinary one-way ANOVA with Tukey’s correction for multiple comparisons, ***P=0.0007 for [1,1] vs. [0,0], ^ns^*P*=0.5151 for [0,1] vs. [0,0], ^ns^*P*=0.9717 for [1,0] vs. [0,0]. **D)** Human ovarian cancer cell lines (ES-2, OAW-42, CaOV-3, and OVCAR8), a hepatocarcinoma cell line (Huh7), and a cervical cancer cell line (Hela) were transduced with both input viruses and the output virus with GFP output ([1,1]). After two days, cells were fixed for flow cytometry analysis. The mean GFP fluorescent intensity is shown. Mean ± s.d.; N=3; unpaired two-tailed t-test, ***P=0.001 for ES-2, **P=0.0015 for CaOV-3.

To design the input modules for the sensing circuit, we used two SPECS, S(*E2F1*)P and S(*cMyc*)P^29^, which were previously demonstrated to be specific to the OVCAR8 ovarian cancer cell line^29^, to transcribe the endoribonuclease Cys4 gene (Input 1) and a novel miRNA-based self-inhibitory gene module (Input 2), respectively (Fig. 2A). The miRNA-based gene module is made of two separate exons for a fusion protein (GAD), consisting of a GAL4 DNA binding domain and the transcriptional transactivator VP16^34^, flanking a synthetic miRNA sequence (miR1)^29,35^, a triplex linker that enables the stabilization of polyA-less mRNAs^36^, a 28bp Cys4 binding site, and a downstream miR1 binding site sequence (Fig. 2A). We defined four different states for the synthetic circuit according to the input status of each module (Sup. Fig. 1): no inputs ([0,0]), one of Input 1 (S(*E2F1*)P / Cys4; [1,0]) or Input 2 (S(*cMyc*)P / miR1; [0,1]), or both inputs ([1,1]). All four of these circuit states incorporate the output module, which drives expression of the output protein (GFP for optimization experiments; TEV protease for detection experiments) only when triggered by both inputs ([1,1]) (Fig. 2A). In the [1,0] state, only Cys4 is expressed, and thus no output protein is generated. On the other hand, with only Input 2 ([0,1]), the miRNA binds to the miRNA binding sites after transcription, inhibits the translation of GAD, and thus prevents the transcription of the output protein. In the absence of any input ([0,0]), there is no active expression of the output protein, due to the absence of the transcriptional activator GAD. However, when both Input 1 and Input 2 are present ([1,1]), the AND-gated circuit is activated, since the transcribed Cys4 (Input 1) binds to the Cys4 binding site on the Input 2 module (Fig. 2A). Cys4 binding releases the inhibitory effect of miR1 by cleaving its binding sites from the GAD mRNA and thus enabling GAD translation (Fig. 2A, Sup. Fig. 1). GAD subsequently binds to the GAL4 promoter (GAL4p) in the output module, driving the conditional expression of a heterologous output protein (GFP for optimization experiments; TEV protease for detection experiments).

We reasoned that the performance of this synthetic circuit would depend on how efficiently each module was delivered and expressed, and how accurately the input modules could sense the transcriptional landscape of transduced cancer cells. To streamline initial characterization of the circuit’s input sensing performance, we used a membrane GFP as output to simplify the analysis process. All four different cellular states (no input ([0, 0]), only one input ([1, 0], or [0, 1]), or both inputs ([1, 1])) were tested by transducing OVCAR8 cells *in vitro* with different combinations of lentiviruses carrying individual circuit components (Fig. 2B). Cells were then fixed for immunofluorescence imaging and flow cytometry analysis to quantify the fraction of GFP-expressing cells and the mean fluorescent intensity of the population. At a multiplicity of infection (MOI) of approximately 5 for each virus, all the cells in the [1, 1] state expressed GFP (data not shown), indicating that the synthetic circuit could be turned on in OVCAR8 cells. To avoid signal saturation caused by over infection, which may complicate the assessment of the synthetic circuit’s performance, we purposely infected the cells with a lower virus titer for each virus. As expected, cells in the [0,0] or [1,0] states displayed no GFP expression (Fig. 2B–C). On the other hand, about 6% of cells in the [0,1] state and 22% of cells in the [1,1] state were GFP positive (Fig. 2B, Sup. Fig. 2). The 6% GFP positivity in the [0,1] state points to leaky transcription of the Input 2 cassette. Cells in the [1,1] state also had the highest mean fluorescent intensity, about 3.5 folder higher than that of the [0,1] state (Fig. 2C). The non-zero frequency of GFP+ cells observed in the [0,1] state-treated cells further supports the hypothesis that the transcription of the Input 2 gene (GAD) was not entirely inhibited by the miRNA repressor module, and that further optimization is required to eliminate this leakiness. Nevertheless, the percent of GFP+ cells detected in the [0,1] state was significantly lower than that detected in the [1,1] state (Fig. 2C, *P*=0.0236).

Next, we sought to demonstrate the ability of the synthetic circuit to distinguish different cancer cell lines. The SPECS in the circuit were designed to bind with ovarian cancer-associated transcription factors enriched in OVCAR8 cells and could putatively be selected for particular input signals relevant to different disease applications. We have previously shown that these SPECS are highly active in OVCAR8 cells, but not in normal ovarian epithelium cells^29^. To test whether these SPECS were specific to OVCAR8 relative to a select subset of cancer cell lines, we infected three additional ovarian cancer lines (ES-2, CaOV3 and OAW-42), a hepatocarcinoma cell line (Huh7), and a cervical cancer cell line (HeLa), with the input and output viruses ([1, 1]). There was no detectable GFP expression in OAW-42, Huh7, or HeLa cells, and negligible GFP expression in ES-2 and CaOV3 cells (Fig. 2D, *P*=0.0001 for ES-2, *P*=0.0015 for CaOV3; Sup. Fig. 2). Altogether, these data suggest that the synthetic circuit was specific for the OVCAR8 cancer line relative to the cell lines assayed, highlighting the power of synthetic biology to tune circuit behavior based on specific cell phenotypes.

### Protease reporter module for amplified response and readout

Having confirmed that the input modules could specifically regulate the output of a fluorescent reporter protein, we set out to develop and validate the TEV protease-based response module. Because of the signal amplification properties of enzyme catalysis, we reasoned that measurements of TEV protease *activity*, rather than abundance, could yield highly sensitive readouts. To that end, two assays were developed to measure TEV protease activity *in vivo* or *ex vivo* (Fig. 1C). Both detection assays utilized similar designs as our previously developed protease-responsive systems^11,16^. For the first assay, we engineered an exogenously administered nanoparticle capable of measuring TEV protease activity *in vivo* to release reporters that can be detected in the urine^11,16,23–25^. A 40 kDa eight-arm poly(ethylene glycol) (PEG-8_40kDa_) nanoparticle was conjugated with a TEV peptide substrate^37^ bearing a ligand-encoded exogenous reporter (Fig. 1C). The average size of these nanoparticles was about 20 nm, as characterized by transmission electron microscopy imaging (Sup. Fig. 3), larger than the size limit of renal filtration. Following systemic administration, the nanosensor is disassembled in response to TEV protease activity, releasing ligand-encoded reporter peptides small enough to renally clear and accumulate in urine. Urinary reporter concentrations are then detected via immunoassay^23^. In parallel, we also developed a detection assay to measure TEV protease activity *ex vivo* in the blood. To this end, we designed a fluorogenic peptide reporter carrying a TEV cleavage site and measured TEV activity in the blood by monitoring fluorescence increase over time (Fig. 1C).

**Figure 3.**
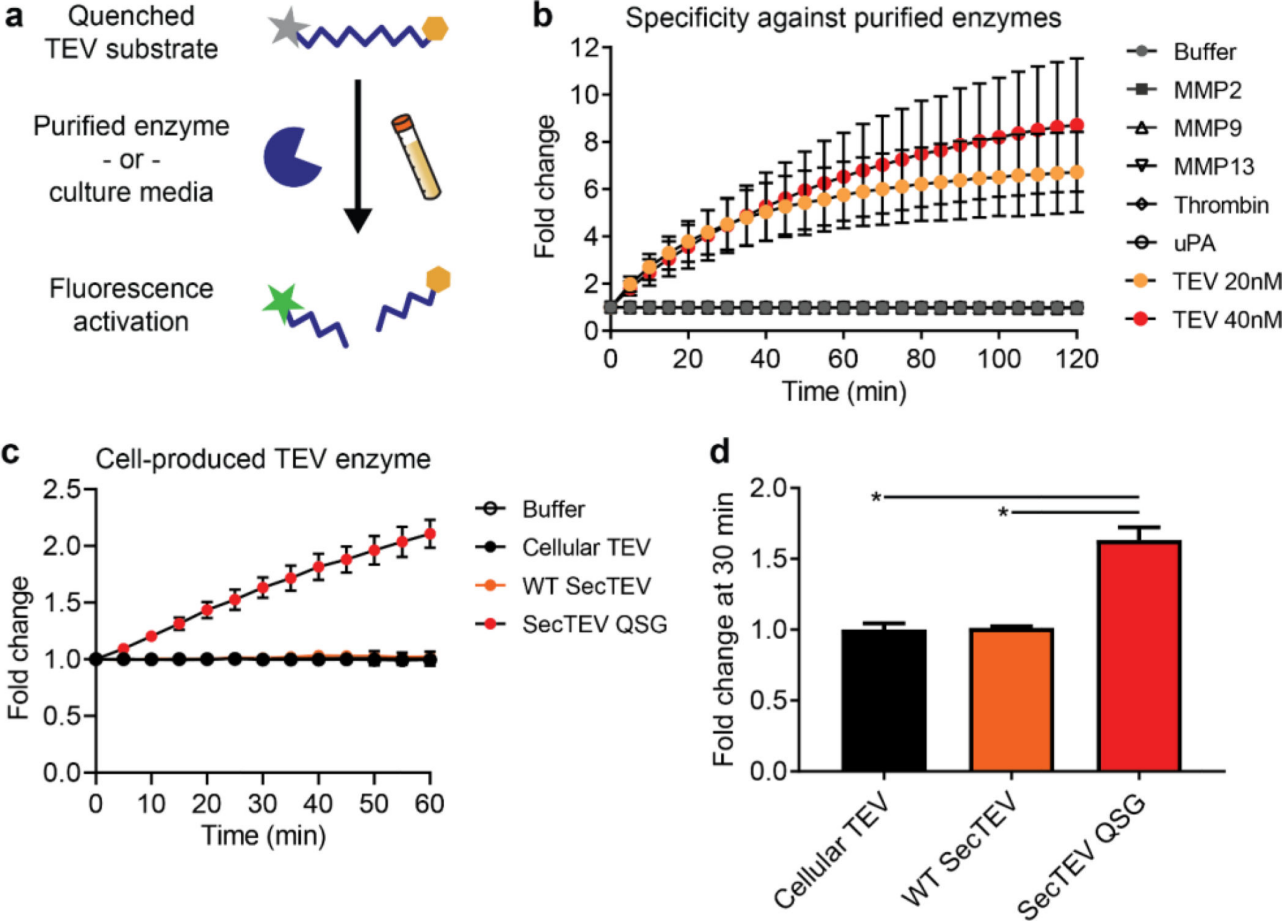
Characterization of the activity-based output signal. **A)** Purified protease enzymes or culture media from mammalian cells recombinantly expressing TEV protease were screened against a FRET-paired TEV substrate, and fluorescence activation was monitored over time. **B)** Kinetic fluorescence curves are shown for 1μM FRET-paired reporter incubated with TEV (20nM or 40nM) as well as MMP2, MMP9, MMP13, thrombin, and uPA (all 20 nM), or buffer without protease (Buffer). Mean ± s.d.; N=3. **C, D)** Activity of cellular wild-type TEV, secreted wild-type TEV (WT SecTEV), and secreted, unglycosylated TEV (SecTEV QSG), present in cell culture media, against FRET-paired TEV substrate, showing cleavage kinetics (C) and fluorescence fold changes at 30 minutes (D). Mean ± s.d.; N=2; unpaired two-tailed t-test, *P=0.0123 SecTEV QSG vs. Cellular TEV, *P=0.0106 for SecTEV QSG vs. WT SecTEV.

We first assessed the function of our TEV peptide substrate *in vitro* by incubating a FRET-based sensor with purified enzymes or with cell-secreted TEV present in conditioned culture media (Fig. 3A). First, we measured cleavage of the FRET-based reporter by monitoring fluorescence signal over time following incubation with purified enzymes, and found that fluorescence intensity increased over time only in the presence of recombinant TEV protease, but not other proteases (Fig. 3B). Next, we tested whether TEV protease maintained its cleavage activity following recombinant expression by mammalian cells. As TEV protease is usually expressed intracellularly, we inserted a N-terminal secretory signal peptide to produce a secreted version of the protein. The secreted wild-type TEV protease (WT SecTEV), however, was not active, as it did not activate the fluorogenic reporter (Fig. 3C). Previous studies indicated that loss of catalytic activity can be attributed to the glycosylation process in the mammalian secretory pathway, and mutations of N23Q, C130S, and T173G in the TEV protease might help the secreted protease regain its activity^33^. Thus, we mutated the protease at the indicated amino acid residues and measured the mutant’s cleavage activity with the fluorogenic detection assay (Fig. 3C). Quantification of fluorescence fold changes for each TEV variant revealed the mutant TEV protease produced and secreted by mammalian cells (SecTEV QSG) successfully cleaved the fluorogenic TEV substrate *in vitro*, while cellular TEV and secreted wild-type TEV did not (Fig. 3D, *P*=0.0123 SecTEV QSG vs. Cellular TEV, *P*=0.0106 for SecTEV QSG vs. WT SecTEV). Based on these characterizations, SecTEV QSG was chosen as the output reporter enzyme for all subsequent studies.

### In vitro validation of the integrated sense-and-respond circuit

**Figure 4.**
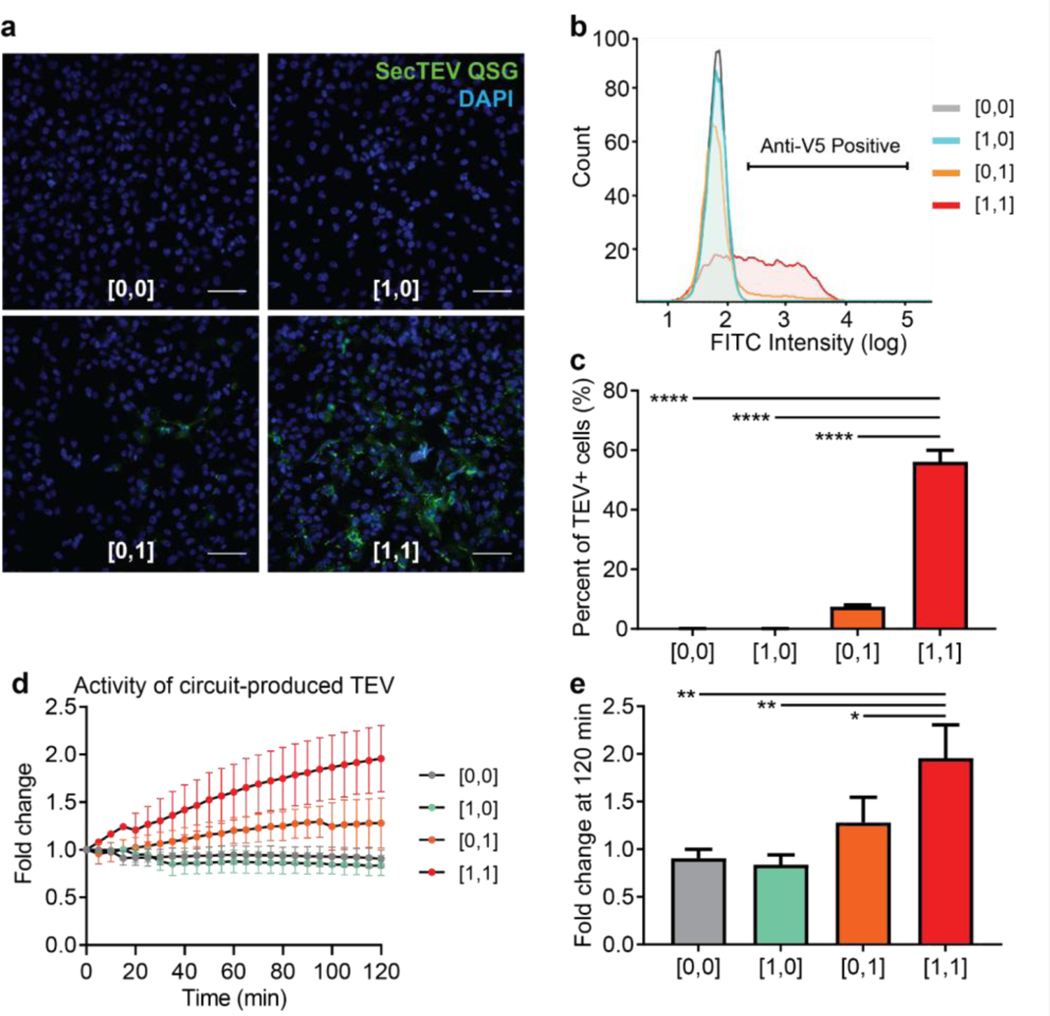
Validation of the sense-and-respond system *in vitro*. OVCAR8 cells were transduced either with the output virus carrying the TEV protease expression cassette ([0,0]), or the output virus with either of two input viruses ([1,0], [0,1]), or the output virus and both of the input viruses ([1,1]). After two days, cells were fixed to measure the TEV protein output, either by immunofluorescence imaging or flow cytometry analysis via detection of the V5 epitope tag. **A)** Representative images of TEV abundance (green; SecTEV QSG) in OVCAR8 cells with different input and output configurations. Slides were counterstained with DAPI (blue). Scale bar: 100μm. **B)** Flow cytometry analysis for V5, with which the TEV protein is tagged, in OVCAR8 cells transduced with different input and output configurations. **C)** Quantification of the percent of transduced OVCAR8 cells positive for TEV, as measured by anti-V5 signal intensity. Mean ± s.d.; N=3; ordinary one-way ANOVA with Tukey’s correction for multiple comparisons, ****P<0.0001. **D, E)** Culture medium of circuit-transduced OVCAR8 cells was collected to measure the activity of circuit-outputted TEV in various input and output configurations. Activity was monitored as increase in fluorescence signal over time (D) as measured by the FRET-based reporter and quantified using fluorescence fold change at 120 minutes (E). Mean ± s.d.; N=3; ordinary one-way ANOVA with Tukey’s correction for multiple comparisons, **P=0.0022 for [1,1] vs. [0,0], **P=0.0015 for [1,1] vs. [1,0], *P=0.0284 for [1,1] vs. [0,1].

Having validated the input (sensing) and output (response) portions of the synthetic circuit individually, we next tested whether the integrated circuit system could detect OVCAR8-associated transcriptional changes *in vitro*. Cultured OVCAR8 cells were transduced with various combinations of input viruses as in Fig. 2B and 2C, as well as with the output virus carrying the SecTEV QSG expression cassette. In this case, the SecTEV QSG cassette was further modified to express TEV protease tagged with the epitope V5 to facilitate immunodetection of the produced TEV protease. Two days post viral transduction, cells were fixed and permeabilized for immunofluorescence imaging or flow cytometry analysis via detection with anti-V5 antibody. Similar to our characterizations using membrane GFP as the output, V5-labelled SecTEV QSG was not detected in conditions transduced with state [0, 0] or [1, 0] lentiviruses (Fig. 4A–C), and low levels were measured for state [0,1]. In contrast, state [1, 1]-transduced cells were positive for SecTEV QSG via immunostaining for V5, as measured by both immunofluorescence imaging (Fig. 4A) and flow cytometry (Fig. 4B; Sup. Fig. 4). Indeed, the [1,1]-transduced population exhibited a significantly greater percentage of TEV+ cells relative to all other state configurations (Fig. 4C). Specifically, 55% of cells transduced with state [1, 1] viruses expressed the TEV protease, based on V5 antigen expression, as compared to approximately 7% of cells exposed to state [0, 1] inputs (Fig. 4C). We defined the ON-OFF ratio for the input AND gate as the relative fold change in output mean intensity in the [1,1] state relative to [0, 1]. The ON-OFF ratio for the SecTEV QSG output was 6 (Sup. Fig. 4), with the output performance of this miRNA-based self-inhibitory AND gate on par with that of other previously reported circuits^29,35^.

Next, we analyzed the system’s sense-and-respond performance by testing whether the activity of SecTEV QSG produced by circuit-transduced OVCAR8 cells could be monitored using the *in vitro* TEV-activity assay. To this end, we harvested the conditioned supernatant from transduced OVCAR8 cells in each of the four different states, and measured TEV protease activity using the fluorogenic reporter. Increased fluorescence signal was observed in supernatant from [1,1] OVCAR8 cells following incubation with the fluorogenic TEV reporter, indicating production and secretion of active TEV by the AND-gate sense-and-respond circuit (Fig. 4D). Modest, non-zero activity of [0,1]-transduced cells was also observed, again suggesting leaky expression of the Input 2 cassette. Quantification of fluorescence fold changes demonstrated that the [1,1] state yielded significantly greater TEV output activity relative to the [0,1] state (Fig. 4E, *P*=0.0284). Collectively, these results demonstrated that our integrated sense-and-respond system could generate OVCAR8-specific output expression, and that output activity levels could be easily detected *in vitro*.

### *In vivo* tumor detection through activity-based readouts in the urine or blood

**Figure 5.**
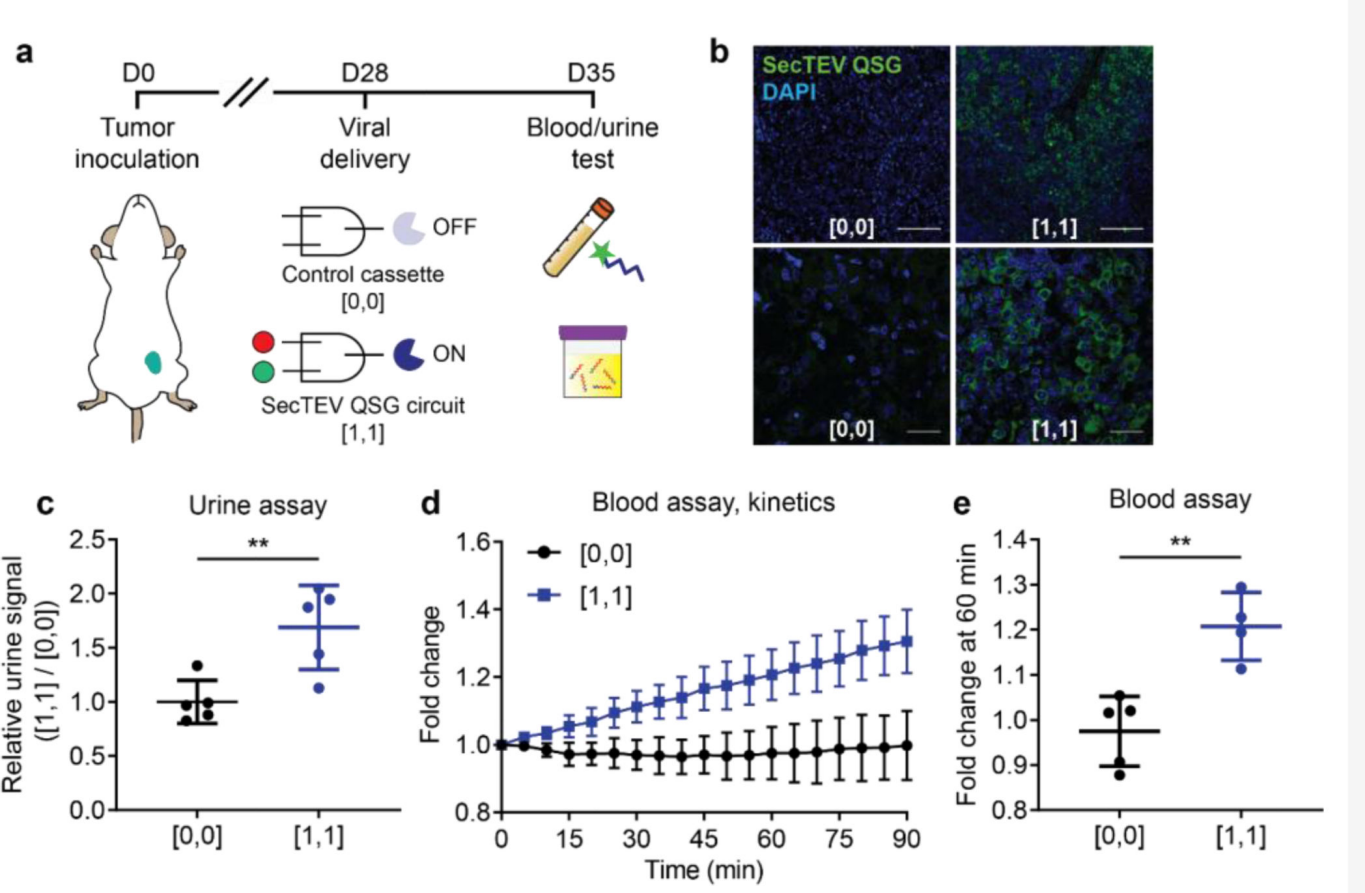
Signal generation via activity-based readout from *in vivo* synthetic circuit. **A)** OVCAR8 tumor-bearing mice were delivered either the output virus bearing the control cassette alone ([0,0]) or the complete sense-and-respond circuit (SecTEV QSG circuit, [1,1]). One week post viral delivery, the TEV-sensitive *in vivo* nanosensor was injected i.v., and urine was collected 1.5 hours post injection. In parallel, blood was drawn for the *ex vivo* readout of TEV activity via the FRET-based reporter. **B)** Abundance of TEV protease (green; SecTEV QSG) in tumor sections from mice transduced with either the output virus alone ([0,0], left) or the complete sense-and-respond circuit ([1,1], right). Scale bar: 100 μm for upper panels, 20 μm for lower panels. **C)** Signal-to-noise ratio ([1,1] / [0,0]) of reporter concentration in the urine collected from mice delivered viral vectors defining the [0,0] and [1,1] states. Mean ± s.d.; N=5 mice per group; unpaired two-tailed t-test, **P=0.0078. **D, E)** Kinetics (D) and fluorescence fold change (E) of TEV activity in the blood from OVCAR8 tumor-bearing mice, measured *ex vivo* by fluorescence activation of FRET-paired reporter. Mean ± s.d.; N=5 mice for [0,0] group, N=4 mice for [1,1] group; unpaired two-tailed t-test, **P=0.0027.

Following successful *in vitro* validation, we sought to apply the integrated sense-and-respond system for *in vivo* ovarian cancer detection using circuit readout assays in either urine or blood. A mouse model of disseminated ovarian cancer was generated by seeding OVCAR8 cells through intraperitoneal (i.p.) injection. Tumor-bearing mice were i.p. injected either with only the V5-tagged SecTEV QSG output virus ([0,0]) or with both input viruses and the output virus ([1,1]) (Fig. 5A). To test whether the heterologous enzyme biomarker SecTEV QSG was being expressed in tumor cells, tumor nodules were resected and stained for V5. Tumor sections from mice infected with both input viruses and the output virus ([1,1]) revealed detectable TEV protein, while there was no positive staining in the control, output virus-only ([0,0]) group (Fig. 5B). Approximately 10% of all resected tumor nodules from the [1,1] group were positive for at least some TEV protease staining, and furthermore most cells in a representative TEV-positive nodule exhibited detectable TEV levels (Fig. 5B). Furthermore, minimal TEV protease staining was observed in tissues surrounding the tumor, such as in the liver, intestine, or other stromal tissues (Sup. Fig. 5). Having verified that outputted TEV protease could be detected within tumor nodules, we also assessed whether this heterologous reporter enzyme was also secreted into circulation. In mice with tumors that stained positively for TEV, TEV protein was also detected in the serum, indicating that the output protease was secreted by transduced cells and entered circulation (Sup. Fig. 6). These results show that i.p. lentiviral circuit administration was sufficient for effective OVCAR8 transduction *in vivo*, and drove expression and secretion of TEV protease by integrating input signals from two OVCAR8-SPCES via a synthetic Boolean AND gate.

Upon confirmation of the output protein expression both in tumor cells and in circulation, we next evaluated whether TEV protease activity could be detected in tumor-bearing, circuit-transduced mice to produce a readout of disease state. To this end, we tested the performance of our two nanotechnology-based detection assays in live animals. For *in vivo* tumor detection experiments, OVCAR8 tumor-bearing mice transduced with the synthetic sensing circuit ([1,1]) or control cassette ([0,0]) were intravenously injected with TEV-responsive nanosensors one week post-circuit transduction. Urine was collected from mice 1.5 hours post injection, and an ELISA-based detection assay was run to detect the presence of liberated reporters^23^. In parallel, blood was collected, and serum samples were reacted with the fluorogenic reporter for the *ex vivo* readout. Urine samples from tumor-bearing mice transduced with the complete circuit ([1,1]) exhibited significantly increased urinary reporter signal relative to tumor-bearing mice transduced with the output virus alone ([0,0]) (Fig. 5C, *P*=0.0078). Similarly, using the fluorogenic reporter in the *ex vivo* detection assay, TEV protease activity was observed in blood samples from mice in the [1,1] group (Fig. 5D), revealing significantly increased circulating TEV activity relative to the [0,0] control group (Fig. 5E, *P*=0.0027). Taken together, these results provide a proof-of-concept demonstration that a synthetic gene circuit can be coupled with nanoparticle-based readout tools to sense ovarian cancer-associated signals *in vivo* in mice and to generate signal-amplified readouts, which are easily traceable in blood and urine.

## Discussion

In this work, we demonstrated a proof-of-principle for an integrated sense-and-respond system, comprised of a genetically-encoded sensing circuit and nanotechnology-based detection tools, for tumor-specific expression of heterologous biomarkers and demonstrated its application in an ovarian cancer model. The AND-gate synthetic gene circuit was engineered to drive the tumor-specific expression of the heterologous reporter TEV protease in response to transcriptional dysregulation associated with ovarian cancer cells. Intraperitoneal lentiviral circuit delivery effectively triggered TEV protease expression from within tumor cells in a disseminated ovarian cancer mouse model. We then deployed two detection assays for measuring the activity of circuit-produced TEV protease against engineered nanomolecular probes *in vivo* or *ex vivo*. Specifically, we engineered peptide-coated nanoparticles that, following systemic administration, interacted with TEV protease *in vivo* to release reporter molecules detectable in the urine. TEV protease activity was also measured in the blood using fluorogenic molecular probes. As a proof-of-concept demonstration, we validated the behavior of this engineered detection system *in vivo* in an ovarian cancer mouse model, where we showed that circuit-transduced tumor cells produced TEV protease and that the activity of this heterologous biomarker could be measured using either of our detection platforms.

Integrating synthetic biology approaches with nanotechnology tools presents a new framework for developing engineered sensors that can monitor, record, and respond to the levels of biological and environmental cues. Each of these approaches presents complementary advantages. Synthetic biology provides a tunable toolkit that could significantly expand the repertoire of accessible disease biomarkers beyond traditional blood-based biomarkers^30^. For example, synthetic gene circuits could be engineered to measure multiple transcriptional, protein, or metabolic biomarkers of disease^2^, and integrate these measurements via logic gating^38–40^ or more complex computations^41,42^. Such circuits could be designed to in turn generate cell-state specific expression of heterologous biomarkers, such as reporter enzymes or effector proteins. By exploiting the unique properties that emerge at the nanoscale, multifunctional nanoparticles could be engineered to target and penetrate disease sites, either to detect the presence or activity of circuit-generated biomarkers or to sense orthogonal signals to multiplex the inputs to the synthetic circuit. Nanosystems have been extensively used to produce both *in vivo* imaging signals^22,43^ and *ex vivo* readouts in accessible biofluids or tissues^14^. Tuning similar nanoparticle sensors towards circuit-produced outputs could provide multiple levels of disease specificity and sensitivity that could ultimately yield highly accurate engineered detection systems.

Here, we demonstrated successful circuit-driven transcriptional sensing as well as output generation *in vivo*, showing both that TEV protease could be expressed in tumor cells and that its activity could be measured by two downstream detection platforms. However, the complete AND-gate synthetic circuit was only validated against a control circuit carrying only the output TEV expression cassette, a key limitation to this study. Circuit performance could be more completely characterized *in vivo* via comparison of the [1,1] state relative to the [0,1] state, the latter of which showed basal expression and activity which can likely be attributed to circuit leakiness. To fully validate the diagnostic ability of this system, it will be critical to assess circuit behavior in syngeneic mouse models using mouse ovarian cancer SPECS as inputs, to evaluate its specificity for ovarian cancer relative to healthy tissues and cancers of other tissues of origin, and to determine its limit of detection via longitudinal studies in tumor-bearing mice. From these studies, analysis of the classification power of this integrated sense-and-respond system will enable assessment of its utility for disease detection. Additionally, we note that the signal generated by our integrated sense-and-respond system is related to the delivery and transduction efficiency of the synthetic gene circuit. In this study, three viruses carrying each genetic component (each of two input and one output modules) were used for delivery. Due to the AND-gate control of the sensing circuit, output expression requires successful genomic integration of all three constructs within each individual cell. Indeed, we observed a relatively low output expression rate (5–10%) within tumor cells, in line with the expectation that the output expression rate would be lower than the transduction rate of each individual virus. To address this limitation, multiple genetic components could be integrated within the genome of a single virus using emerging gene therapy approaches^19^. Different delivery modalities, such as intratumoral viral injection or tumor-targeted delivery of the viral vectors^18,19^, could also be explored to enhance the transduction rate. The performance of our system could further be improved by optimizing the stringency and dynamic range of the promoters controlling the AND gate components^44^ or by incorporating engineered TEV protease variants with optimized catalytic activity^45^. Lastly, validation of the proposed integrative approach using different gene circuits, input signals, output biomarkers, and disease models will be critical to establish its general utility.

In summary, we present the design and proof-of-concept application of an engineered, multi-component biosensor that integrates synthetic genetic circuits with nanotechnology tools for tumor detection. Our platform is highly tunable and can generate amplified signals for easy readouts in either the blood or urine following *in vivo* deployment. Moreover, with simple design modifications, such as replacing the SPECS inputs from ovarian-cancer SPECS to SPECS for other tumor types, our system could be adapted to detect additional tumor types. Finally, expression of outputs that activate cytotoxic loads linked to nanoparticles could be potentially implemented as a safe and effective cancer therapy. We envision that the integration of synthetic biology and nanotechnology proposed by this work may enable new classes of engineered sense-and-respond systems.

## Materials and Methods

All experimental procedures were performed in accordance with the Guide for the Care and Use of Laboratory Animals of the National Institutes of Health. The protocol was approved by the Institutional Animal Care and Use Committee (IACUC) of the Massachusetts Institute of Technology.

### Antibodies and recombinant proteins

The following primary antibodies were used in this study: mouse anti-V5 antibody (ThermoFisher, R960–25), rabbit anti-V5 antibody (Sigma, V8137), Alexa Fluor 555 donkey-anti-mouse (ThermoFisher, A-31570), Alexa Fluor 488 chicken-anti-mouse (ThermoFisher, A-21200), goat anti-mouse serum albumin (Abcam, ab19194), mouse anti-rabbit HRP (Santa Cruz, SC-2357), mouse anti-goat HRP (Santa Cruz, SC-2354), goat anti-mouse HRP (Santa Cruz, SC-2005), rabbit anti-FITC (GeneTex, GTX26644), streptavidin-conjugated HRP (Abcam, ab7403).

The following recombinant proteins were used in this study: TEV protease (Ananspec, AS-72227), MMP2 (RD Systems, 902-MP-010), MMP9 (RD Systems, 911-MP-010), MMP13 (RD Systems, 511-MM-010), uPA (RD Systems, 1310-SE-010), thrombin (Haematologic Technologies, HCT-0020).

### Plasmids

All synthetic circuit plasmid maps are provided in Supplementary Figures 7–10. The sequences for relevant biological parts are provided in Supplementary Table 1. The synthetic circuit plasmids were constructed by conventional restriction enzyme cloning and Gibson assembly (Gibson Assembly Master Mix, NEB, E2611) with the backbone of lentiviral vector FUGW (Addgene, 14883). The three synthetic promoters S(*E2F1*)P, S(*cMyc*)P, and GAL4BS were designed as previously described^29^. Specifically, for the inputs, the synthetic promoter S(*E2F1*)P was placed upstream of the 5’ end of Cys4, while S(*cMyc*)P was placed upstream of the 5’ end of the RNA-based self-inhibitory gene cassette. The RNA-based self-inhibitory gene component consisted of two exons of GAD, a miRNA sequence, a triplex sequence, a 28bp Cys4 binding sequence, and a miRNA binding site sequence. For the output plasmid, synthetic promoter GAL4BS was placed upstream of transmembrane GFP (hTFR-GFP, Addgene plasmid 45060) or TEV protease (pcDNA3.1-V5-hTEV, Addgene plasmid 65800). The TEV protease gene was modified by inserting an IgK leader sequence at the N-terminus for secretion (cloned from vector pSecTag2 B, ThermoFisher, V90020), a V5 tag sequence at the 5’ end, and a Myc tag sequence at the 3’ end. To make active secreted TEV protease, we created a N23Q, C130S, T173G triple TEV mutant by site-directed mutagenesis (QuikChange Site-Directed Mutagenesis Kit, Agilent, 200519), using the following primers:

N23Q, 5’-ctgccacctcacccaggagtccgacggcc-3’;

C130S, 5’-tggggattcacagcgctagcaattttggaaataccaataattactttacatcc-3’;

T173G, 5’-ggtgtccgatacaagcagcacattcccatccag-3’.

### Cell culture and viral production

All cell lines in this study were maintained in Dulbecco’s modified Eagle medium (DMEM; Invitrogen, 11965092) containing 10% fetal bovine serum (ThermoFisher, 10437028) and antibiotics (25U/ml penicillin and 25 μg/ml streptomycin), and cultured in humidified, 5% CO_2_ environment at 37°C.

Lentiviruses were produced by co-transfecting HEK293T cells with 6μg lentiviral expression vector, 4.5μg delta 8.9 vector, and 3μg VSVG packaging vector in a 10cm dish using a calcium phosphate transfection kit (CalPhos Mammalian Transfection kit, Clontech, 631312). Two days post-transfection, the supernatant was harvested, centrifuged at 3000 rpm for 10 minutes, and snap frozen in liquid nitrogen.

### In vitro viral transduction and synthetic circuit characterization

To validate the behavior of the synthetic circuit, OVCAR8 ovarian cancer cells were seeded in 96 or 6-well plates, and cells were transduced with the output virus ([0,0]), output virus with one of two input viruses ([1,0], [0,1]), or both input viruses and the output virus ([1,1]) in the presence of 4 μg/ml polybrene (Santa Cruz, SC-134220). 8 hours post transduction, cells were replenished with fresh DMEM medium containing 10% fetal bovine serum and 1% penicillin/streptomycin and were cultured for an additional two days. Cells in 96-well plates were directly fixed with 4% paraformaldehyde (PFA, EMS, 50–980-495) for immunofluorescence imaging, while cells in 6-well plates were trypsinized and then fixed with 4% PFA for flow cytometry analysis. For the GFP output cassette, fixed cells were directly processed for immunofluorescence imaging or flow cytometry. However, for the V5-tagged SecTEV QSG output cassette, cells were first permeabilized with 0.1% Triton X-100 (Sigma Aldrich) in PBS for 5 minutes, blocked in 3% bovine serum albumin in PBS (PBSA) for 30 minutes, and incubated with mouse anti-V5 antibody (1:500, ThermoFisher, R960–25) diluted in 3% PBSA for 1.5 hours. Cells were washed three times in PBS every 5 minutes, and then incubated with Alexa Fluor 555 donkey anti-mouse secondary antibody (1:1000 dilution, ThermoFisher, A-31570) or Alexa Fluor 488 chicken-anti-mouse secondary antibody (1:1000 dilution, ThermoFisher, A-21200) for 1 hour. Following staining, cells were imaged on a Nikon microscope for immunofluorescence imaging, or analyzed on a LSRII Fortessa cytometer (BD Biosciences) for flow cytometry. Flow cytometry data analysis was performed in FlowJo (TreeStar Inc, Ashland, OR).

### TEV activity sensors and in vitro measurement assays

For the fluorogenic TEV reporter, the TEV specific peptide substrate Glu-Asn-Leu-Tyr-Phe-Gln-Gly was labeled with 5-FAM and QXL520 quencher (AnaSpec, AS-72227). The fluorogenic TEV substrate was incubated with recombinant enzyme according to manufacturers’ protocols. Proteolytic cleavage of this quenched substrate was quantified by increases in fluorescence signal intensity (Ex/Em = 490/520 nm) over time as measured by fluorimeter (Tecan Infinite M200 Pro).

For synthesis of TEV-responsive nanoparticles, 40 kDa, eight-arm multivalent (poly)ethylene glycol (PEG) molecules with maleimide reactive handles (JenKem Technology) were dissolved in PBS and filtered through a 0.2μm membrane filter (Acrodisc). Filtered PEG nanoparticles were reacted with at least 20-fold excess of cysteine-terminated TEV-specific peptide for at least 1 hour at room temperature. The TEV-specific peptide (Sequence: Biotin-eGvndneeGffsar-K(FAM)-dGGENLYFQGGGC) consists of a glutamate fibrinopeptide B (GluFib) urinary reporter, encoded with an N-terminal biotin and 5-FAM on the opposite terminus^23^, and a TEV-specific cleavage site, and was synthesized by the Koch Institute Polymers and Peptides Core at MIT. Following the maleimide-cysteine coupling, unconjugated peptides were removed from the nanoparticle complexes through centrifugal filtration with 30kD spin column filters (Millipore, UFC503096). The size of synthesized PEG nanoparticles was measured by transmission electron microscopy (TEM) in with 2% uranyl acetate on an FEI Tecnai Spirit Transmission Electron Microscope at the Koch Institute facility.

To measure the activity of cell-secreted TEV *in vitro*, OVCAR8 cells were seeded and transduced as previously described. Cells were cultured in OptiMEM (Gibco, 11058021) overnight, and supernatant was collected. 100 μl of collected supernatant was incubated with fluorogenic TEV substrate (1 μM), and proteolytic cleavage was monitored by increase in fluorescence intensity over time as measured by fluorimeter.

### ELISA for ligand-encoded reporters

96-well plates (Nunc) were coated with capture antibodies (Rabbit anti-FITC, GeneTex, 1:1000 dilution) for one hour in coating buffer (0.05M carbonate bicarbonate, pH 9.6), and blocked with 3% (w/v) bovine serum albumin (BSA) in PBS for 30 minutes. After incubating with 100 μl sample solution for 1 hour at room temperature or overnight at 4 °C, the wells were washed with PBST (PBS with 0.2% Tween) five times, and incubated with detection antibody (Strepavidin-HRP, Abcam, 1:3000 dilution) in PBST for 1 hour before adding the chromogenic substrate TMB (ThermoFisher, 34028). Oxidation of TMB for 5–10 minutes allowed quantification of reporter concentrations. For urine experiments, samples were diluted at 1:300 in 3% BSA in PBS.

### Ovarian cancer mouse model and in vivo TEV activity measurements

All animal studies were approved by the Massachusetts Institute of Technology (MIT) committee on animal care (MIT protocol 0420–023-23) and conducted in compliance with ARRIVE guidelines. To generate the disseminated ovarian cancer mouse model, three to four-week-old female NCr nude mice (Taconic) were injected with 5 million OVCAR8 cells intraperitoneally (i.p.). OVCAR8 cells were modified to stably express luciferase, allowing us to monitor tumor growth through intravital IVIS imaging (PerkinElmer, Koch Institute Animal Imaging and Preclinial Testing Core, MIT). For imaging, 100 μl of 15mg/ml D-luciferin potassium salt (GoldBio, LUCK-100) dissolved in PBS was injected into each mouse subcutaneously, and luciferase activity was measured after 5 minutes.

Four weeks after seeding of OVCAR8 cells, mice were intraperitoneally injected with different combinations of lentivirus mix (corresponding to the [0,0] or [1,1] state) diluted in 100 μl sterile PBS. After one week, 30 μl blood was collected from each mouse through tail vein bleeding and mixed with 30 μl PBS containing 1 μM EDTA (Invitrogen, 15575020). The collected blood was centrifuged at 13000rpm for 2 minutes, and the supernatant plasma was collected. For the fluorogenic detection assay, plasma samples were incubated with fluorogenic TEV peptide substrate (1 μM), and substrate cleavage was monitored by fluorescence increase over time as measured by a Tecan plate reader.

To detect cancer through urinary readouts, tumor-bearing mice were transduced with lentivirus combinations as described above. One week post-transduction, mice were injected intravenously with TEV-responsive nanosensors (1 μM in 200 μl sterile PBS) and subcutaneously with 500 μl of sterile PBS to facilitate urinary production. One hour post nanosensor injection, mice were placed in custom housing with a 96-well plate base for urine collection. After 30 minutes, bladders of the mice were voided to collect urine. Collected urine samples were diluted 300-fold for ELISA measurement.

### Histology

Immunohistochemistry was performed to determine whether transduced tumor cells expressed TEV protease. OVCAR8 tumor-bearing mice were transduced with lentivirus combinations as previously described, and tumor nodules were extracted and fixed in 4% PFA in PBS overnight. Fixed tissue was paraffin embedded and sectioned for immunostaining (Histology Core, Koch Institute, MIT). Sections were stained with mouse anti-V5 antibody (1:1000 dilution, ThermoFisher, R960–25) and appropriate secondary antibody, and images were acquired by a Nikon microscope.

## Supplementary Material

Supplement

Additional results and figures including: inputs and states of the AND gate; output and specificity characterization of the synthetic circuit; characterization of the PEG-based nanosensor; *in vitro* characterization of circuit ON-OFF ratio; circuit-produced TEV abundance in healthy tissues; detection of circuit-produced TEV protease in the blood; plasmid maps for circuit components; sequences of biological parts.

Inputs and states of the RNA/Csy4 based AND gate; output and specificity characterization of the synthetic circuit; characterization of the PEG-based nanosensor; *in vitro* characterization of circuit ON-OFF ratio; circuit-produced TEV abundance in healthy tissues; detection of circuit-produced TEV protease in the blood following *in vivo* transduction.

## Data Availability

The data that support the findings of this study are available upon request to sbhatia@mit.edu.

## References

[1] Weber W; Fussenegger M. Emerging Biomedical Applications of Synthetic Biology. Nature Reviews Genetics. Nature Publishing Group January 29, 2012, pp 21–35. 10.1038/nrg3094. PMC7097403 22124480

[2] Brophy JAN; Voigt CA Principles of Genetic Circuit Design. Nature Methods 2014, 11 (5), 508–520. 10.1038/nmeth.2926. 24781324 PMC4230274

[3] Khalil AS; Collins JJ Synthetic Biology: Applications Come of Age. Nature Reviews Genetics. Nature Publishing Group May 2010, pp 367–379. 10.1038/nrg2775. PMC2896386 20395970

[4] Kwon EJ; Lo JH; Bhatia SN Smart Nanosystems: Bio-Inspired Technologies That Interact with the Host Environment. Proceedings of the National Academy of Sciences 2015, 112 (47), 14460–14466. 10.1073/pnas.1508522112. PMC4664363 26598694

[5] Park SM; Aalipour A; Vermesh O; Yu JH; Gambhir SS Towards Clinically Translatable in Vivo Nanodiagnostics. Nature Reviews Materials 2017, 2 (May). 10.1038/natrevmats.2017.14. PMC5985817 29876137

[6] Soleimany AP; Bhatia SN Activity-Based Diagnostics: An Emerging Paradigm for Disease Detection and Monitoring. Trends in Molecular Medicine 2020, 26 (5), 450–468. 10.1016/j.molmed.2020.01.013. 32359477 PMC8290463

[7] Dudani JS; Warren AD; Bhatia SN Harnessing Protease Activity to Improve Cancer Care. Annual Review of Cancer Biology 2018, 2 (1), 353–376. 10.1146/annurev-cancerbio-030617-050549.

[8] Slomovic S; Pardee K; Collins JJ Synthetic Biology Devices for in Vitro and in Vivo Diagnostics. Proceedings of the National Academy of Sciences of the United States of America 2015, 112 (47), 14429–14435. 10.1073/pnas.1508521112. 26598662 PMC4664311

[9] Danino T; Prindle A; Kwong GA; Skalak M; Li H; Allen K; Hasty J; Bhatia SN Programmable Probiotics for Detection of Cancer in Urine. Science Translational Medicine 2015, 7 (289), 289ra84. 10.1126/scitranslmed.aaa3519. PMC4511399 26019220

[10] Aalipour A; Chuang HY; Murty S; D’Souza AL; Park S. min; Gulati, G. S.; Patel, C. B.; Beinat, C.; Simonetta, F.; Martinić, I.; Gowrishankar, G.; Robinson, E. R.; Aalipour, E.; Zhian, Z.; Gambhir, S. S. Engineered Immune Cells as Highly Sensitive Cancer Diagnostics. Nature Biotechnology 2019, 37 (5), 531–539. 10.1038/s41587-019-0064-8. PMC7295609 30886438

[11] Kirkpatrick JD; Warren AD; Soleimany AP; Westcott PMK; Voog JC; Martin-Alonso C; Fleming HE; Tammela T; Jacks T; Bhatia SN Urinary Detection of Lung Cancer in Mice via Noninvasive Pulmonary Protease Profiling. Science Translational Medicine 2020, 12 (537), eaaw0262. 10.1126/scitranslmed.aaw0262. 32238573 PMC7894603

[12] Lakshmanan A; Jin Z; Nety SP; Sawyer DP; Lee-Gosselin A; Malounda D; Swift MB; Maresca D; Shapiro MG Acoustic Biosensors for Ultrasound Imaging of Enzyme Activity. Nature Chemical Biology 2020, 16 (9), 988–996. 10.1038/s41589-020-0591-0. 32661379 PMC7713704

[13] Ronald JA; Chuang HY; Dragulescu-Andrasi A; Horia SS; Gambhira SS Detecting Cancers through Tumor-Activatable Minicircles That Lead to a Detectable Blood Biomarker. Proceedings of the National Academy of Sciences of the United States of America 2015, 112 (10), 3068–3073. 10.1073/pnas.1414156112. 25713388 PMC4364239

[14] Soleimany AP; Kirkpatrick JD; Su S; Dudani JS; Zhong Q; Bekdemir A; Bhatia SN Activatable Zymography Probes Enable In Situ Localization of Protease Dysregulation in Cancer. Cancer Research 2021, 81 (1), 213 LP – 224. 10.1158/0008-5472.CAN-20-2410. 33106334 PMC8244999

[15] Lu TK; Bowers J; Koeris MS Advancing Bacteriophage-Based Microbial Diagnostics with Synthetic Biology. Trends in Biotechnology 2013, 31 (6), 325–327. 10.1016/j.tibtech.2013.03.009. 23608522

[16] Kwong GA; Von Maltzahn G; Murugappan G; Abudayyeh O; Mo S; Papayannopoulos IA; Sverdlov DY; Liu SB; Warren AD; Popov Y; Schuppan D; Bhatia SN Mass-Encoded Synthetic Biomarkers for Multiplexed Urinary Monitoring of Disease. Nature Biotechnology 2013, 31 (1), 63–70. 10.1038/nbt.2464. PMC3542405 23242163

[17] Tastanova A; Folcher M; Müller M; Camenisch G; Ponti A; Horn T; Tikhomirova MS; Fussenegger M. Synthetic Biology-Based Cellular Biomedical Tattoo for Detection of Hypercalcemia Associated with Cancer. Science Translational Medicine 2018, 10 (437), eaap8562. 10.1126/scitranslmed.aap8562. 29669854

[18] Milone MC; O’Doherty U. Clinical Use of Lentiviral Vectors. Leukemia. Nature Publishing Group July 1, 2018, pp 1529–1541. 10.1038/s41375-018-0106-0. PMC6035154 29654266

[19] Dunbar CE; High KA; Joung JK; Kohn DB; Ozawa K; Sadelain M. Gene Therapy Comes of Age. Science. American Association for the Advancement of Science January 12, 2018. 10.1126/science.aan4672. 29326244

[20] Cantore A; Ranzani M; Bartholomae CC; Volpin M; Valle P.della; Sanvito F; Sergi LS; Gallina P; Benedicenti F; Bellinger D; Raymer R; Merricks E; Bellintani F; Martin S; Doglioni C; D’Angelo A; Driessche T. vanden; Chuah MK; Schmidt M; Nichols T; Montini E; Naldini L. Liver-Directed Lentiviral Gene Therapy in a Dog Model of Hemophilia B. Science Translational Medicine 2015, 7 (277), 277ra28–277ra28. 10.1126/scitranslmed.aaa1405. PMC5669486 25739762

[21] Campochiaro PA; Lauer AK; Sohn EH; Mir TA; Naylor S; Anderton MC; Kelleher M; Harrop R; Ellis S; Mitrophanous KA Lentiviral Vector Gene Transfer of Endostatin/Angiostatin for Macular Degeneration (GEM) Study. Human Gene Therapy 2017, 28 (1), 99–111. 10.1089/hum.2016.117. 27710144 PMC5278797

[22] Garland M; Yim JJ; Bogyo M. A Bright Future for Precision Medicine: Advances in Fluorescent Chemical Probe Design and Their Clinical Application. Cell Chemical Biology 2016, 23 (1), 122–136. 10.1016/j.chembiol.2015.12.003. 26933740 PMC4779185

[23] Warren AD; Kwong GA; Wood DK; Lin KY; Bhatia SN Point-of-Care Diagnostics for Noncommunicable Diseases Using Synthetic Urinary Biomarkers and Paper Microfluidics. Proceedings of the National Academy of Sciences of the United States of America 2014, 111 (10), 3671–3676. 10.1073/pnas.1314651111. 24567404 PMC3956200

[24] Kwon EJ; Dudani JS; Bhatia SN Ultrasensitive Tumour-Penetrating Nanosensors of Protease Activity. Nature Biomedical Engineering 2017, 1 (4), 1–10. 10.1038/s41551-017-0054. PMC5621765 28970963

[25] Loynachan CN; Soleimany AP; Dudani JS; Lin Y; Najer A; Bekdemir A; Chen Q; Bhatia SN; Stevens MM Renal Clearable Catalytic Gold Nanoclusters for in Vivo Disease Monitoring. Nature Nanotechnology 2019, 14 (9), 883–890. 10.1038/s41565-019-0527-6. PMC7045344 31477801

[26] Wu MR; Nissim L; Stupp D; Pery E; Binder-Nissim A; Weisinger K; Enghuus C; Palacios SR; Humphrey M; Zhang Z; Maria Novoa E; Kellis M; Weiss R; Rabkin SD; Tabach Y; Lu TK A High-Throughput Screening and Computation Platform for Identifying Synthetic Promoters with Enhanced Cell-State Specificity (SPECS). Nature Communications 2019, 10 (1), 1–10. 10.1038/s41467-019-10912-8. PMC6599391 31253799

[27] Morel M; Shtrahman R; Rotter V; Nissim L; Bar-Ziv RH Cellular Heterogeneity Mediates Inherent Sensitivity-Specificity Tradeoff in Cancer Targeting by Synthetic Circuits. Proceedings of the National Academy of Sciences of the United States of America 2016, 113 (29), 8133–8138. 10.1073/pnas.1604391113. 27385823 PMC4961148

[28] Nissim L; Bar-Ziv RH A Tunable Dual-Promoter Integrator for Targeting of Cancer Cells. Molecular Systems Biology 2010, 6. 10.1038/msb.2010.99. PMC3018173 21179016

[29] Nissim L; Wu MR; Pery E; Binder-Nissim A; Suzuki HI; Stupp D; Wehrspaun C; Tabach Y; Sharp PA; Lu TK Synthetic RNA-Based Immunomodulatory Gene Circuits for Cancer Immunotherapy. Cell 2017, 171 (5), 1138–1150.e15. 10.1016/j.cell.2017.09.049. 29056342 PMC5986174

[30] Hori SS; Gambhir SS Mathematical Model Identifies Blood Biomarker-Based Early Cancer Detection Strategies and Limitations. Science Translational Medicine 2011, 3 (109), 1–10. 10.1126/scitranslmed.3003110. PMC3423335 22089452

[31] Fader AN; Java J; Krivak TC; Bristow RE; Tergas AI; Bookman MA; Armstrong DK; Tanner EJ; Gershenson DM The Prognostic Significance of Pre- and Post-Treatment CA-125 in Grade 1 Serous Ovarian Carcinoma: A Gynecologic Oncology Group Study. Gynecologic Oncology 2014, 132 (3), 560–565. 10.1016/j.ygyno.2013.11.016. 24333362 PMC4390028

[32] Kapust RB; Tözsér J; Fox JD; Anderson DE; Cherry S; Copeland TD; Waugh DS Tobacco Etch Virus Protease: Mechanism of Autolysis and Rational Design of Stable Mutants with Wild-Type Catalytic Proficiency. Protein Engineering, Design and Selection 2001, 14 (12), 993–1000. 10.1093/protein/14.12.993. 11809930

[33] Cesaratto F; López-Requena A; Burrone OR; Petris G. Engineered Tobacco Etch Virus (TEV) Protease Active in the Secretory Pathway of Mammalian Cells. Journal of Biotechnology 2015, 212, 159–166. 10.1016/j.jbiotec.2015.08.026. 26327323

[34] Nissim L; Wu MR; Pery E; Binder-Nissim A; Suzuki HI; Stupp D; Wehrspaun C; Tabach Y; Sharp PA; Lu TK Synthetic RNA-Based Immunomodulatory Gene Circuits for Cancer Immunotherapy. Cell 2017, 171 (5), 1138–1150.e15. 10.1016/j.cell.2017.09.049. 29056342 PMC5986174

[35] Nissim L; Perli SD; Fridkin A; Perez-Pinera P; Lu TK Multiplexed and Programmable Regulation of Gene Networks with an Integrated RNA and CRISPR/Cas Toolkit in Human Cells. Molecular Cell 2014, 54 (4), 698–710. 10.1016/j.molcel.2014.04.022. 24837679 PMC4077618

[36] Ebert MS; Sharp PA MicroRNA Sponges: Progress and Possibilities. RNA. 2010. 10.1261/rna.2414110. PMC2957044 20855538

[37] Waugh DS An Overview of Enzymatic Reagents for the Removal of Affinity Tags. Protein Expression and Purification. Protein Expr Purif December 2011, pp 283–293. 10.1016/j.pep.2011.08.005. PMC3195948 21871965

[38] Bonnet J; Yin P; Ortiz ME; Subsoontorn P; Endy D. Amplifying Genetic Logic Gates. Science 2013, 340, 599–603. 23539178 10.1126/science.1232758

[39] Siuti P; Yazbek J; Lu TK Synthetic Circuits Integrating Logic and Memory in Living Cells. Nature Biotechnology 2013, 31 (5), 448–452. 10.1038/nbt.2510. 23396014

[40] Gao XJ; Chong LS; Kim MS; Elowitz MB Programmable Protein Circuits in Living Cells. Science 2018, 361 (6408), 1252–1258. 10.1126/science.aat5062. 30237357 PMC7176481

[41] Daniel R; Rubens JR; Sarpeshkar R; Lu TK Synthetic Analog Computation in Living Cells. Nature 2013, 497 (7451), 619–623. 10.1038/nature12148. 23676681

[42] Roquet N; Soleimany AP; Ferris AC; Aaronson S; Lu TK Synthetic Recombinase-Based State Machines in Living Cells. Science 2016, 353 (6297), aad8559. 10.1126/science.aad8559. 27463678

[43] Weissleder R; Tung CH; Mahmood U; Bogdanov A. In Vivo Imaging of Tumors with Protease-Activated near-Infrared Fluorescent Probes. Nature Biotechnology 1999, 17 (4), 375–378. 10.1038/7933. 10207887

[44] Brophy JAN; Voigt CA Principles of Genetic Circuit Design. Nature Methods 2014, 11 (5), 508–520. 10.1038/nmeth.2926. 24781324 PMC4230274

[45] Sanchez MI; Ting AY Directed Evolution Improves the Catalytic Efficiency of TEV Protease. Nature Methods 2020, 17 (2), 167–174. 10.1038/s41592-019-0665-7. 31819267 PMC7004888

