# Supplementary material for: Synthetic circuit-driven expression of heterologous enzymes for disease detection
Source: ACS Synth Biol. Author manuscript; Available in PMC 2025 Jul 23. (PMC12285725; doi:10.1021/acssynbio.1c00133)
Supplement: Supplement [file NIHMS2083147-supplement-Supplement.docx]

**Supplementary Information for:** Synthetic circuit-driven expression of heterologous enzymes for disease detection

**Authors:** Jiang He^1,2†^, Lior Nissim^3,4†^, Ava P. Soleimany^1,2,5†^, Adina Binder-Nissim^3,6^, Heather E. Fleming^1,2^, Timothy K. Lu^3,7^, Sangeeta N. Bhatia^1,2,7-11^*

**Affiliations:**

^1^Koch Institute for Integrative Cancer Research, Massachusetts Institute of Technology, Cambridge, MA, 02139, USA.

^2^Harvard–MIT Division of Health Sciences and Technology, Institute for Medical Engineering and Science, Massachusetts Institute of Technology, Cambridge, MA, 02139, USA.

^3^Synthetic Biology Group, Research Laboratory of Electronics, Department of Biological Engineering, Massachusetts Institute of Technology, Cambridge, MA, 02139, USA.

^4^Department of Biochemistry and Molecular Biology, The Institute for Medical Research Israel-Canada, Hadassah Medical School, The Hebrew University of Jerusalem, 91120, Jerusalem, Israel.

^5^Harvard Graduate Program in Biophysics, Harvard University, Boston, MA, 02115, USA.

^6^Department of Family Medicine, Meuhedet Health Maintenance Organization, Tel Aviv, Israel.

^7^Department of Electrical Engineering and Computer Science, Massachusetts Institute of Technology, Cambridge, MA, 02139, USA.

^8^Department of Medicine, Brigham and Women’s Hospital, Harvard Medical School, Boston, MA, 02115, USA.

^9^Broad Institute of Massachusetts Institute of Technology and Harvard, Cambridge, MA, 02139, USA.

^10^Wyss Institute at Harvard, Boston, MA, 02115, USA.

^11^Howard Hughes Medical Institute, Cambridge, MA, 02139, USA.

^†^These authors contributed equally to this work.

*Corresponding author. Email: sbhatia@mit.edu

**
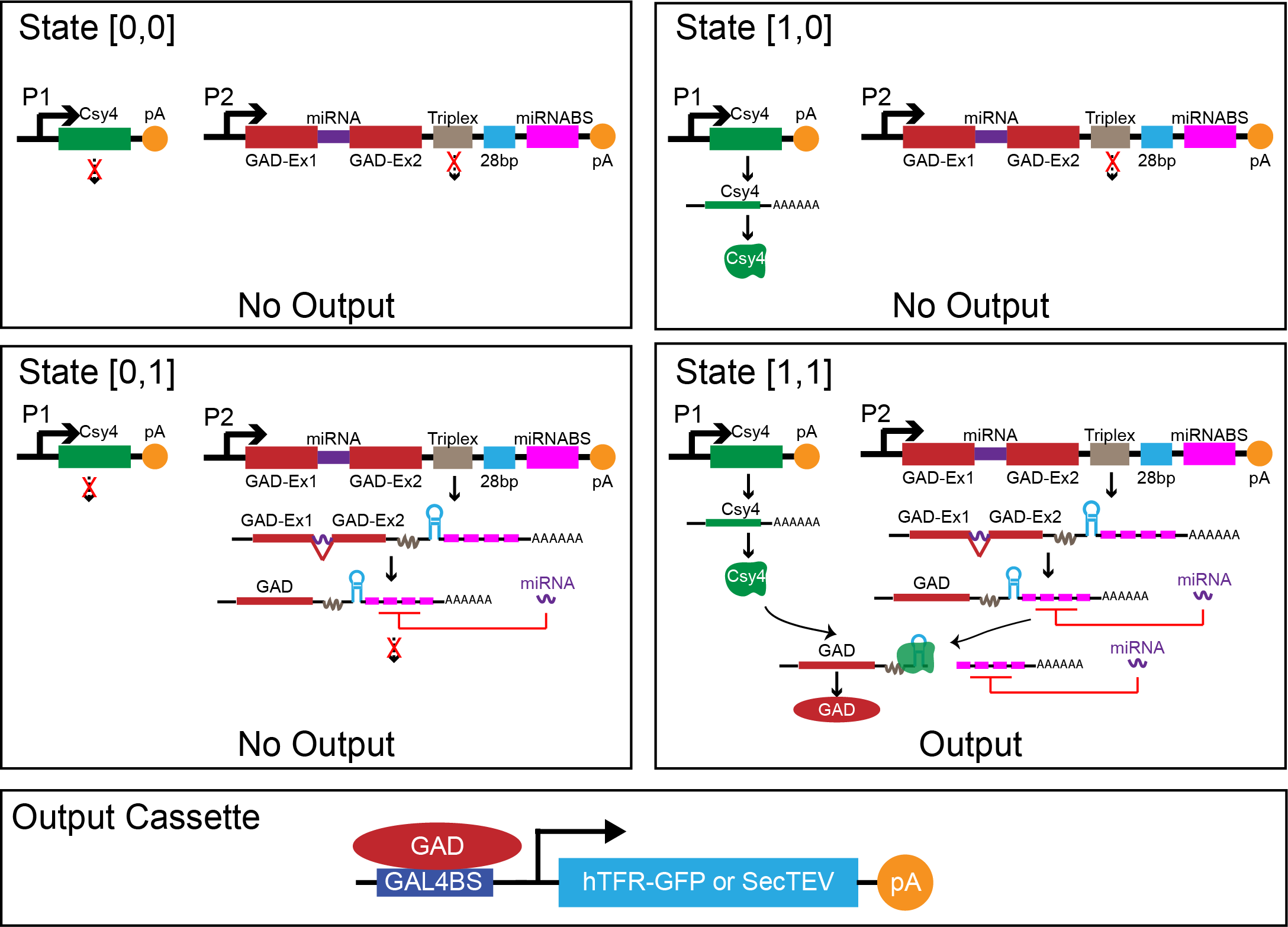
**

**Supplementary Figure 1. Inputs and states of the RNA/Csy4 based AND gate.** In all four states, the output module carrying the output cassette is always present in the circuit. Input states are defined within the square brackets by whether input module 1 and input module 2 are active, where 0 means inactive and 1 means active. In state [0,0], neither P1 (S(*E2F1*)P) nor P2 (S(*cMyc*)P) is active, and thus there is no output. In state [1,0], corresponding to when the transcription factor for S(*E2F1*)P but not for S(*cMyc*)P is present, P1 is active and Csy4 is transcribed. However, as P2 is inactive, no GAD, the fusion protein carrying the GAL4 binding domain and the transcription activator that controls the SPEC on the output expression cassette, will be transcribed; thus, there is no output. In state [0,1], corresponding to when the transcription factor for S(*cMyc*)P but not for S(*E2F1*)P is present, only P2 is active, and GAD will be transcribed. However, due to the inhibitory effect of the miRNA on the GAD transcript, the transcript cannot be translated; thus there is no output. In state [1,1], both P1 and P2 are active. Csy4 will be transcribed to lift the self-inhibitory effect of the miRNA on the GAD transcript, leading to translation of GAD. Only in this state will the output be generated, driven GAD transcriptional activation of the GAL4BS synthetic promoter via active GAL4BD (GAL4 DNA binding domain).

**
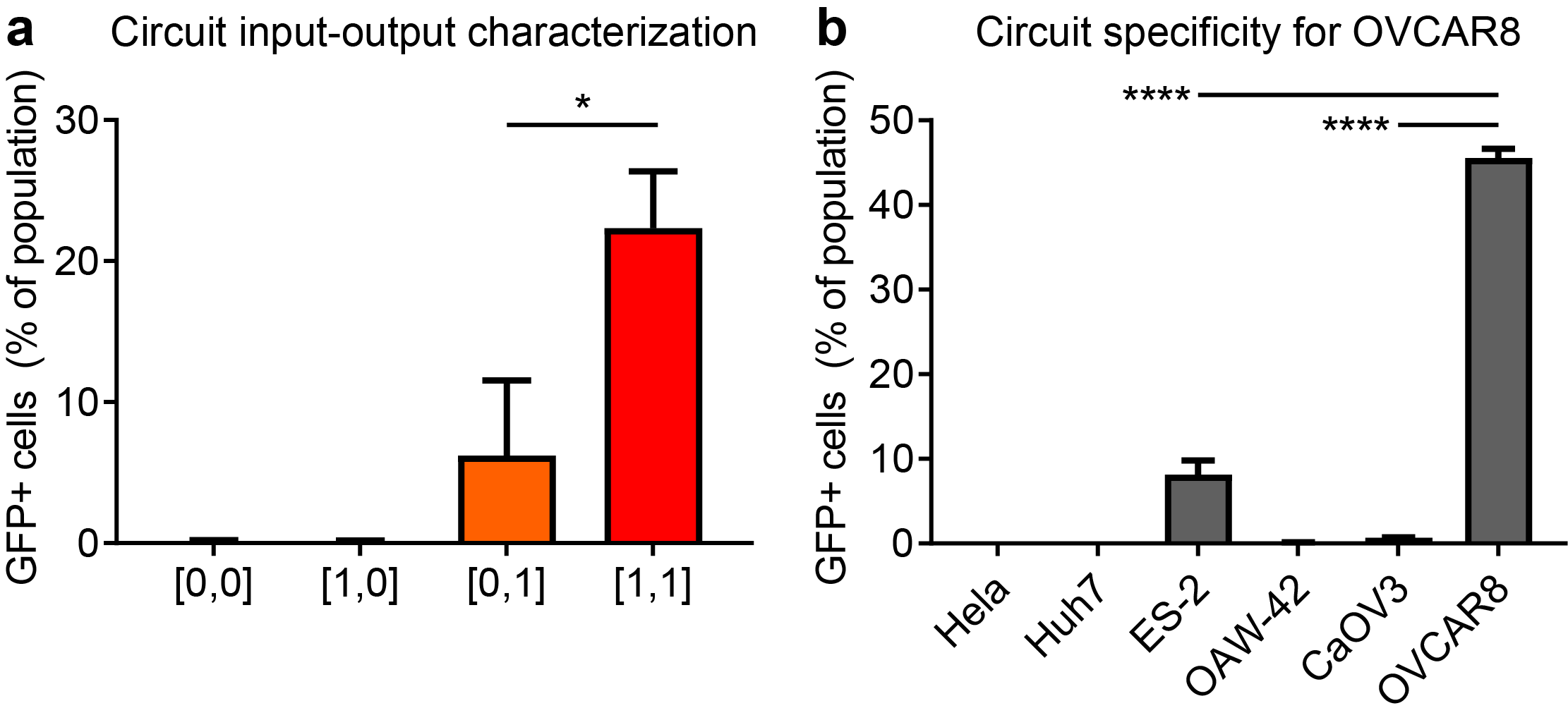
**

**Supplementary Figure 2. Output and specificity characterization of the synthetic circuit. A)** OVCAR8 cells were transduced with the output virus carrying a membrane GFP expression cassette ([0,0]), or the output virus with one of two input viruses ([1,0], [0,1]), or both input viruses and the output virus ([1,1]). After two days, cells were fixed for flow cytometry. Quantification of the percentage of the population positive for GFP for cells with different input and output configurations. Mean ± s.d.; N=3; unpaired two-tailed t-test, **P*=0.0140. **B)** Human ovarian cancer cell lines (ES-2, OAW-42, CaOV-3, and OVCAR8), a hepatocarcinoma cell line (Huh7), and a cervical cancer cell line (Hela) with both input viruses and the output virus with GFP output ([1,1]). After two days, cells were fixed for flow cytometry analysis. Quantification of the percentage of the population positive for GFP for different cell lines. Mean ± s.d.; N=3; unpaired two-tailed t-test, *****P*<0.0001.


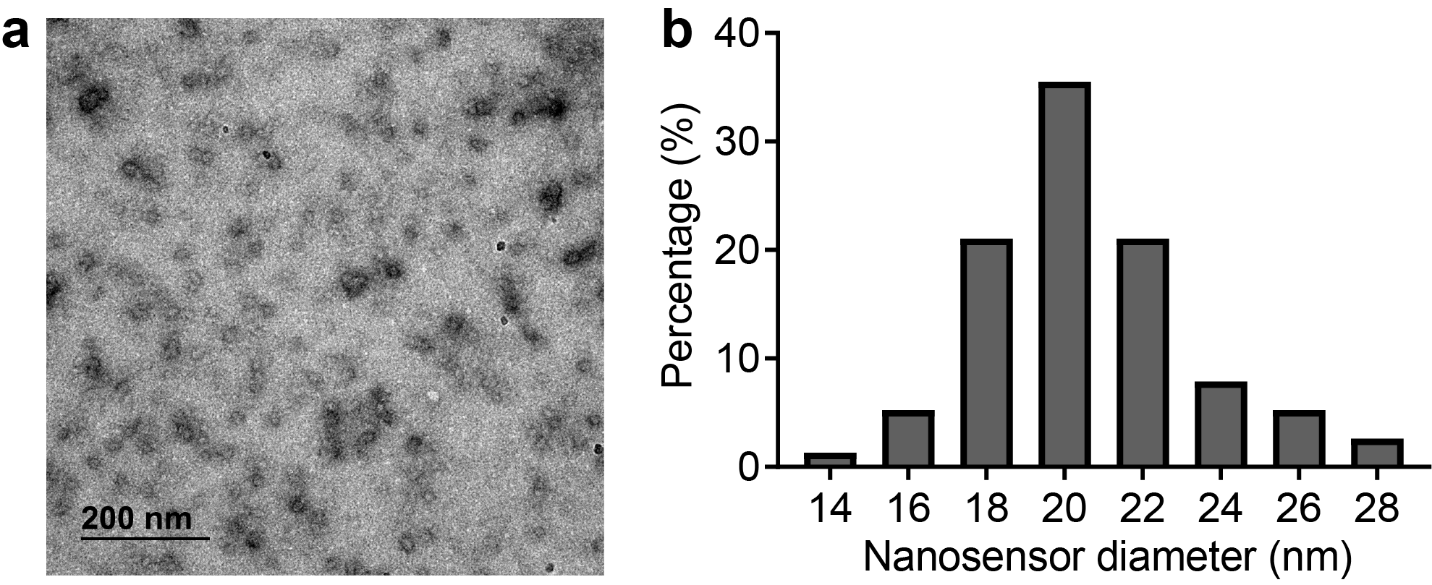


**Supplementary Figure 3. Characterization of the PEG-based nanosensor. A)** TEM image of the TEV-sensitive nanosensor. Scale bar: 200 nm. **B)** The histogram shows results of the size analysis from TEM images of the nanosensors (mean diameter, 20.6 ± 2.8 nm (s.d.), N=76 particles).


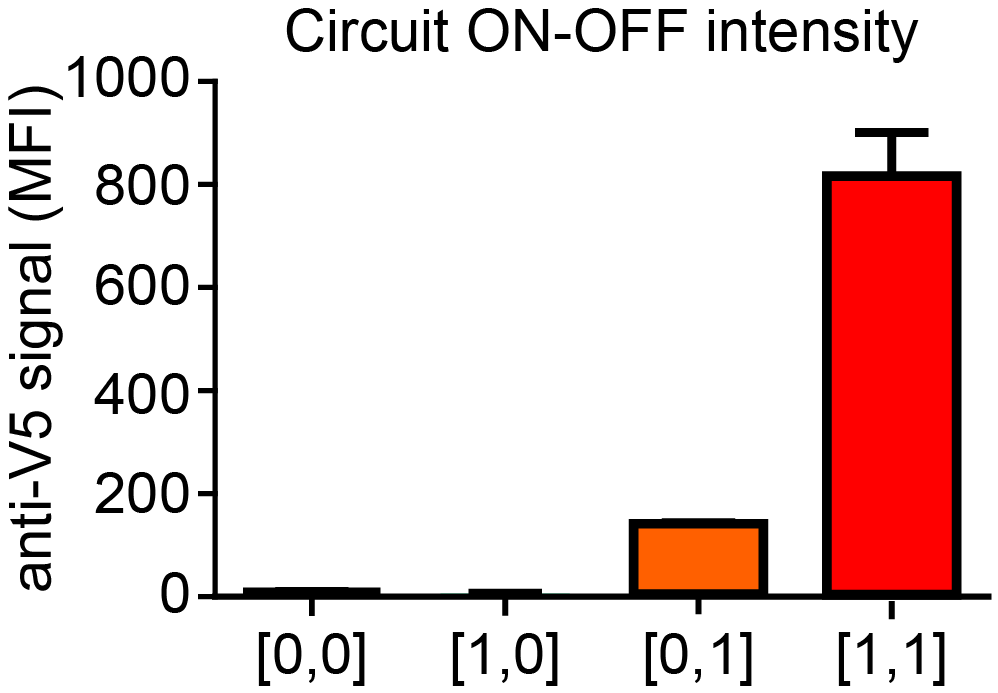


**Supplementary Figure 4. *In vitro* characterization of circuit ON-OFF ratio.** OVCAR8 cells were transduced either with the output virus carrying the TEV protease expression cassette ([0,0]), or the output virus with either of two input viruses ([1,0], [0,1]), or the output virus and both of the input viruses ([1,1]). After two days, cells were fixed to visualize the presence of the TEV protein output by flow cytometry analysis via detection of the V5 epitope tag (Fig. 4b). Quantification of flow cytometry analysis of mean GFP fluorescent intensity with different input and output configurations, as gated by [0,0] state. Mean ± s.d.; N=3.


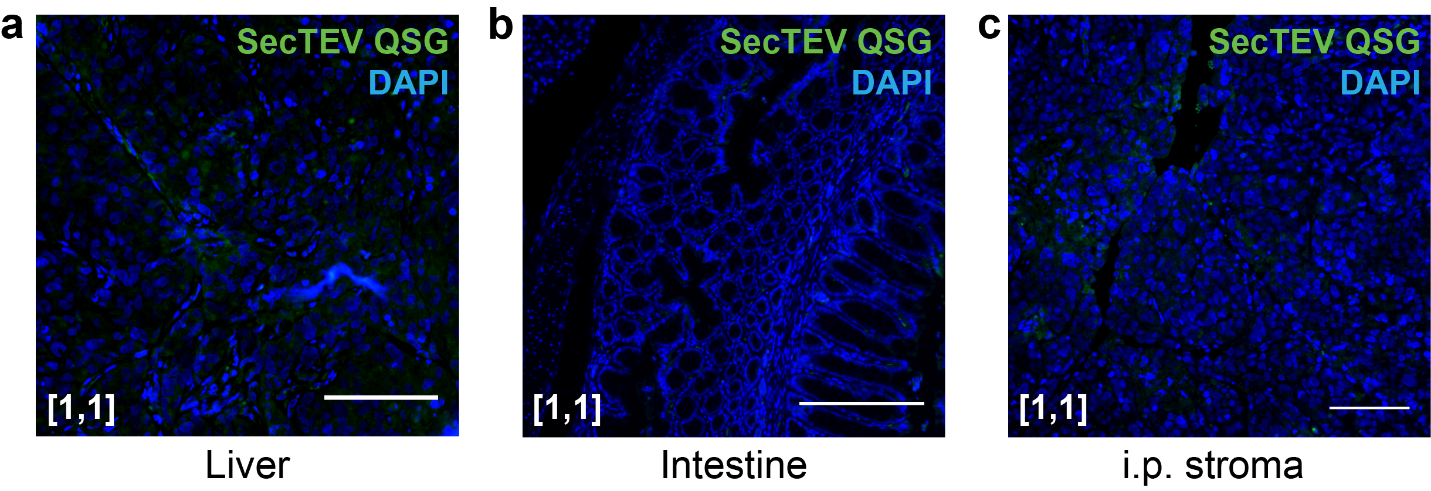


**Supplementary Figure 5. Circuit-produced TEV abundance in healthy tissues.** 3-4 week old nude mice were i.p. injected with 5 million luciferase-expressing OVCAR8 cells. After 4 weeks, mice were i.p. injected with the complete synthetic circuit, corresponding to the [1,1] state. For mice with positive TEV staining in tumor nodules, TEV protease abundance in regions surrounding the tumor, including the liver **(A)**, the intestine **(B)**, and the surrounding intraperitoneal stroma **(C)**, was assessed by staining with an anti-V5 antibody. Scale bar: 100 µm.


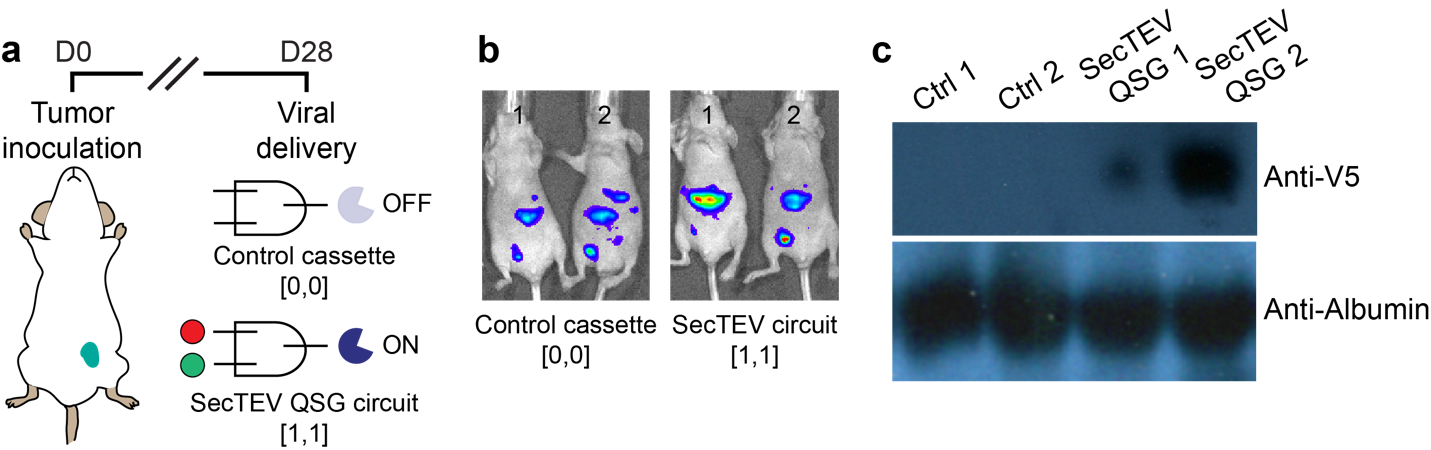


**Supplementary Figure 6. Detection of circuit-produced TEV protease in the blood following *in vivo* transduction. A)** 3-4 week old nude mice were i.p. injected with 5 million luciferase-expressing OVCAR8 cells. After 4 weeks, the mice were i.p. injected with the control cassette alone (Ctrl, [0,0]) or the complete SecTEV QSG synthetic circuit (SecTEV QSG, [1,1]). End stage blood collection was conducted through cardiac puncture two weeks post viral transduction. **B)** Representative IVIS images of OVCAR8-luciferase-inoculated mice transduced with either the control cassette or the complete SecTEV QSG synthetic circuit. **C)** Abundance of secreted TEV protease in the serum from viral-transduced OVCAR8 tumor-bearing mice, as measured via Western blotting against the V5 epitope tag present on circuit-produced SecTEV QSG.


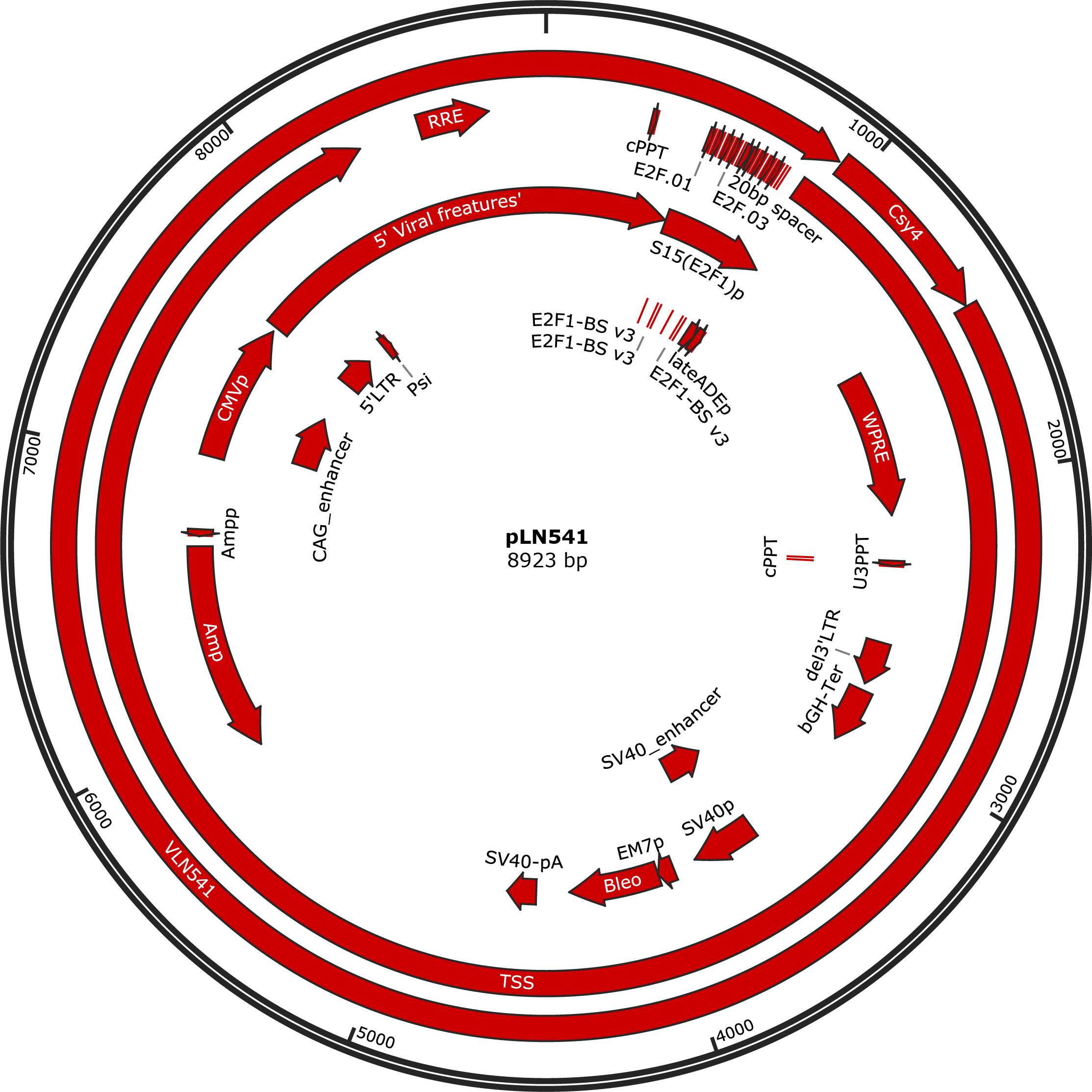


**Supplementary Figure 7. Plasmid map for Input 1 (Csy4) circuit component.**


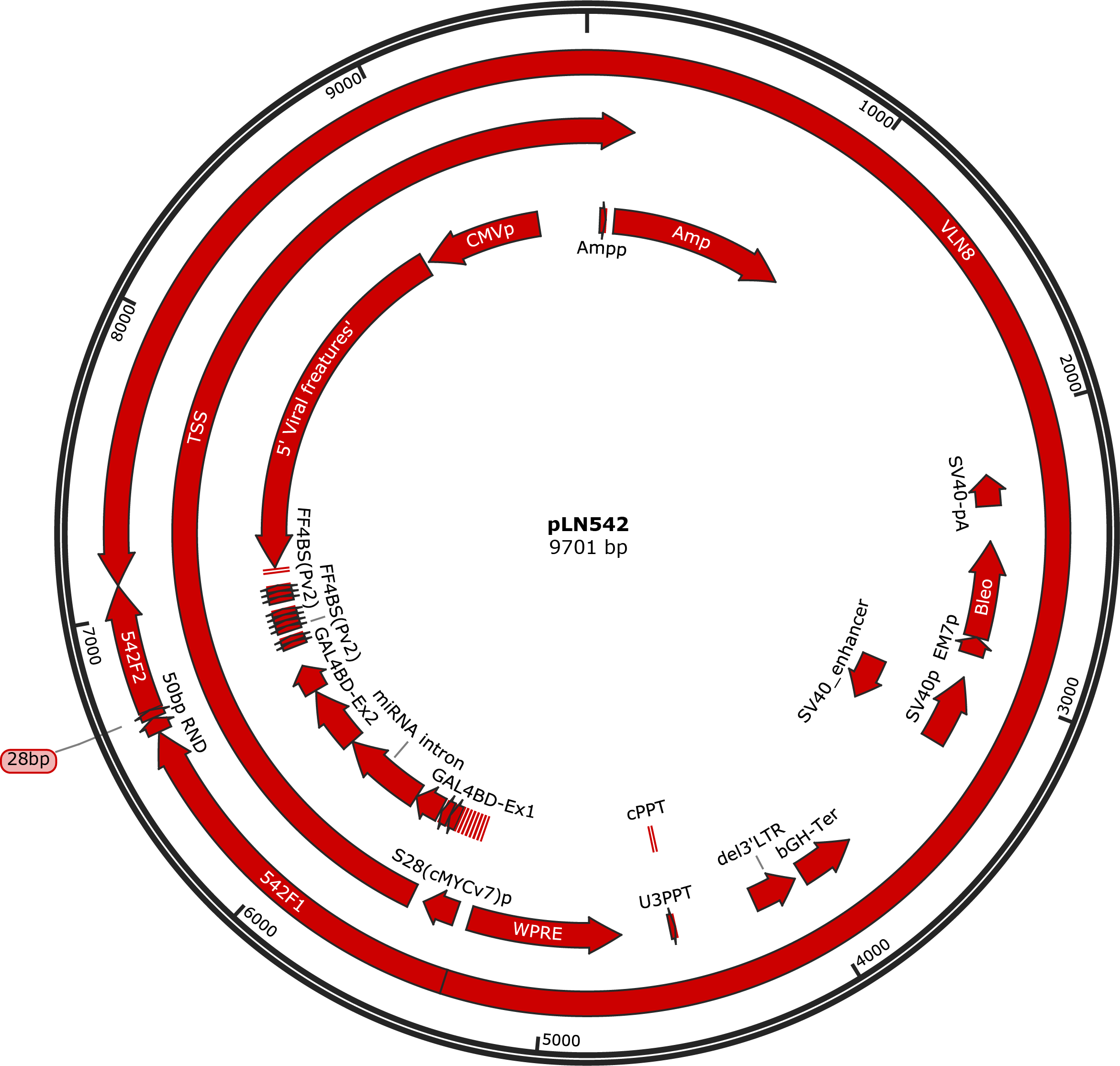


**Supplementary Figure 8. Plasmid map for Input 2 (GAD) circuit component.**


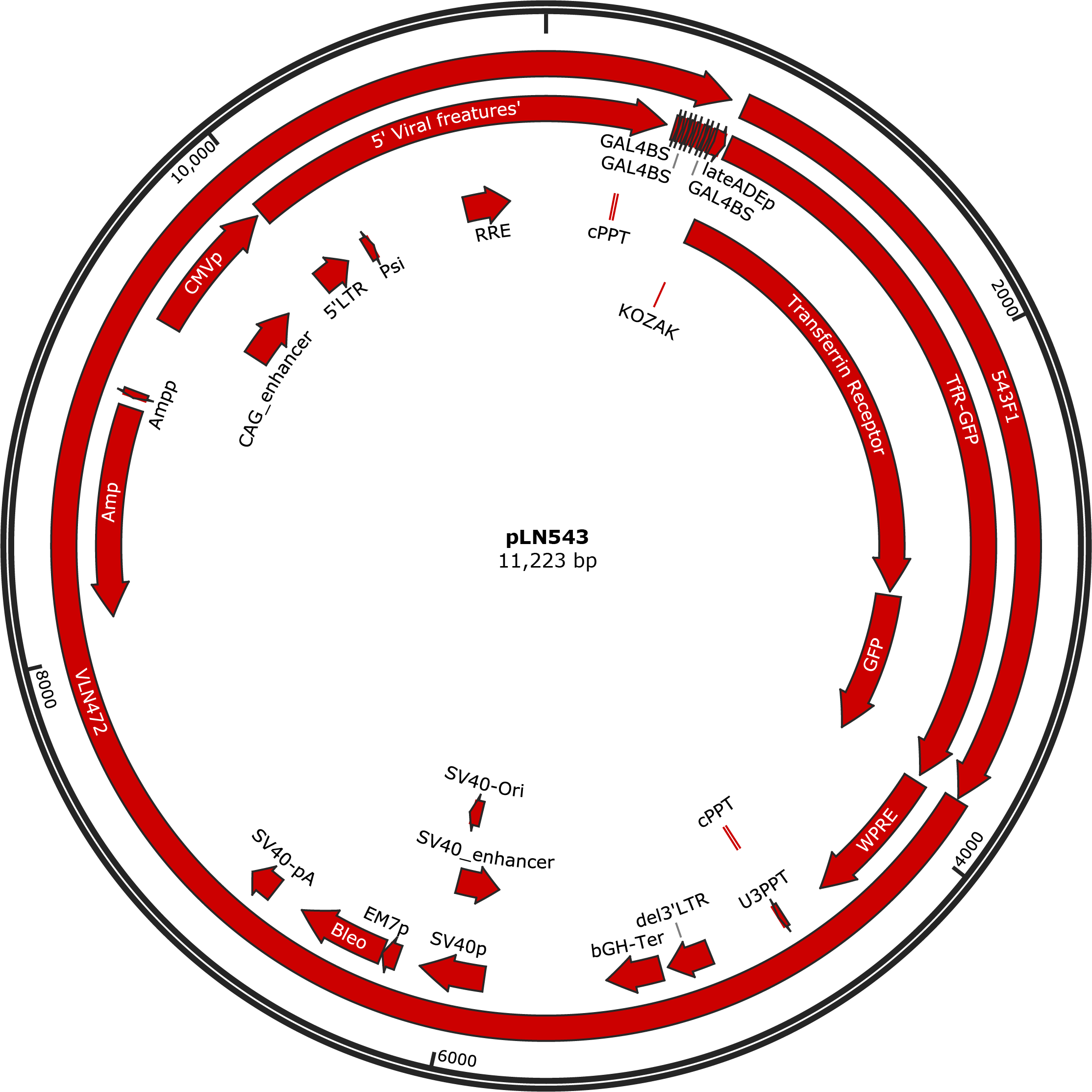


**Supplementary Figure 9. Plasmid map for output cassette with hTFR-GFP.**


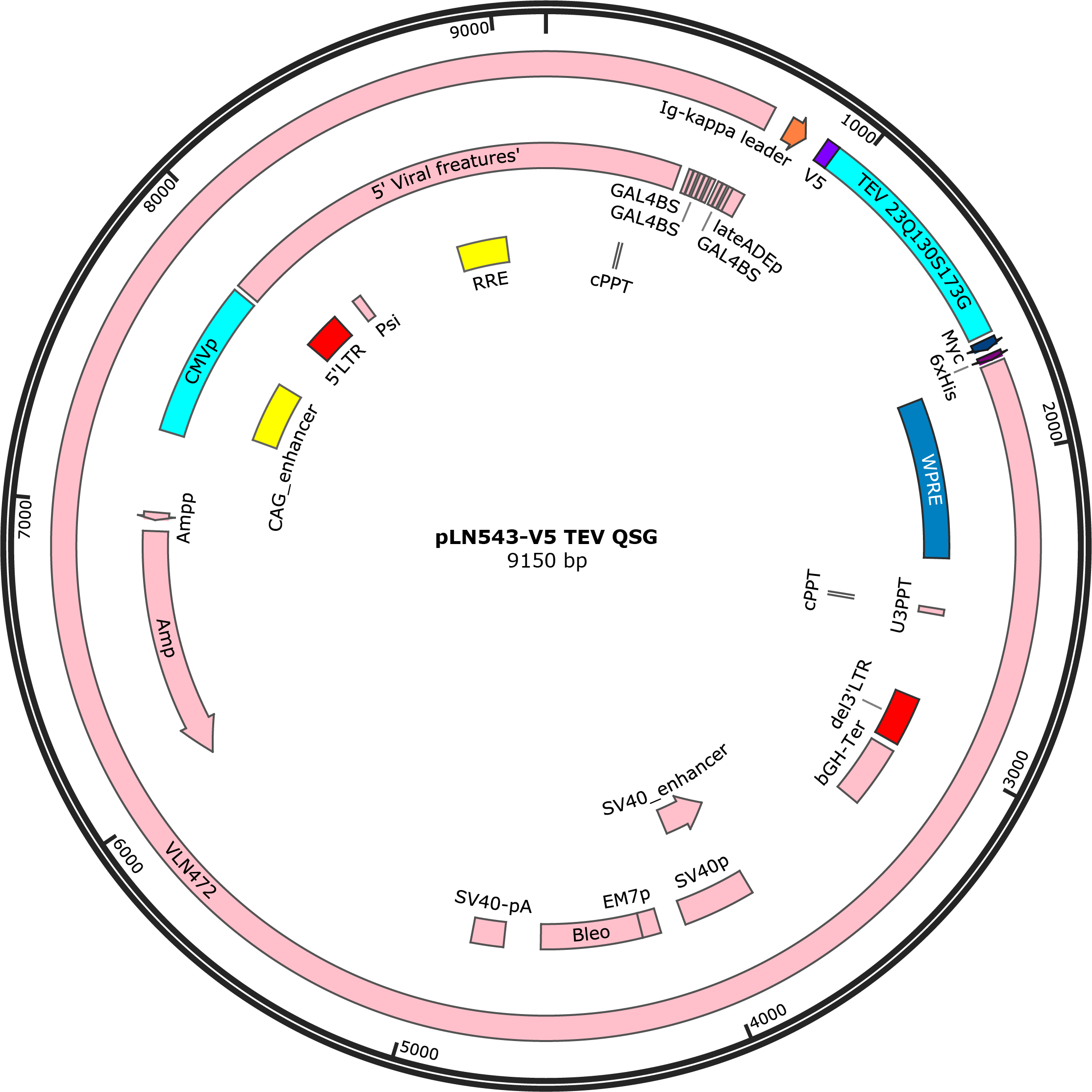


**Supplementary Figure 9. Plasmid map for output cassette with hTFR-GFP.Supplementary Table 1. Plasmid parts and their sequences.**

| **Name** | **Sequence** |
| --- | --- |
| *S(E2F1)P*, promoter | ATAAGCTTGATATCGCGGCCGCCCTGCACCTGCAGGTACGCGCGAAAACTGAGCGCGCCAAAATCGCTGAGCGCGAAACGGACGGGGCGGGAAGACTTACGCGCGAAAACTGGTCGCGCCAAAGAGGCTGAGCGCGAAACGTCCGGGGCGGGAAGCGAATTCTGCCCTACTGACACTGCCTGCCGTACGCGCGAAAACTGAGCGCGCCAAAATCGCTGAGCGCGAAACGGACGGGGCGGGAAGACTTACGCGCGAAAACTGGTCGCGCCAAAGAGGCTGAGCGCGAAACGTCCGGGGCGGGAAGCGAATATCCGCGGCGCGCCAGACGCTAGCGGGGGGCTATAAAAGGGGGTGGGGGCGTTCGTCCTCACTCTAGATCTGCGATCTAAGTAAGCTTGGCATTCCGGTACTGTTGGTAAACCAGCT |
| Csy4 | GACCACTATCTGGACATCAGACTGAGGCCCGATCCTGAGTTCCCTCCCGCCCAGCTGATGAGCGTGCTGTTTGGCAAGCTGCATCAGGCTCTGGTCGCCCAAGGCGGAGACAGAATCGGCGTGTCCTTCCCCGACCTGGACGAGTCCCGGAGTCGCCTGGGCGAGCGGCTGAGAATCCACGCCAGCGCAGACGATCTGCGCGCCCTGCTGGCCCGGCCTTGGCTGGAGGGCCTGCGGGATCATCTGCAGTTTGGCGAGCCCGCCGTGGTGCCACACCCAACACCCTACCGCCAGGTGAGCCGCGTGCAGGCCAAGTCAAATCCCGAGAGACTGCGGCGGAGGCTGATGAGGCGACATGATCTGAGCGAGGAGGAGGCCAGAAAGAGAATCCCCGACACAGTGGCCAGAGCCCTGGATCTGCCATTTGTGACCCTGCGGAGCCAGAGCACTGGCCAGCATTTCAGACTGTTCATCAGACACGGGCCCCTGCAGGTGACAGCCGAGGAGGGCGGATTTACATGCTATGGCCTGTCTAAAGGCGGCTTCGTGCCCTGGTTC |
| *S(cMyc)P*, promoter | GTCCGCCACGTGCGCGACAGTCCGCCACGTGCGCGACTGGTCCGCCACGTGCGCGACCGTCCGCCACGTGCGCGACCGGTCCGCCACGTGCGCGACCGTCCGCCACGTGCGCGACGTGTCCGCCACGTGCGCG |
| GAL4BD Ex1, of GAD | ATGAAGCTACTGTCTTCTATCGAACAAGCATGCGATATTTGCCGACTTAAAAAGCTCAAGTGCTCCAAAGAAAAACCGAAGTGCGCCAAGTGTCTGAAGAACAACTGGGAGTGTCGCTACTCTCCCAAAACCAAAAG |
| GAL4BD Ex2, of GAD | GTCTCCGCTGACTAGGGCACATCTGACAGAAGTGGAATCAAGGCTAGAAAGACTGGAACAGCTATTTCTACTGATTTTTCCTCGAGAAGACCTTGACATGATTTTGAAAATGGATTCTTTACAGGATATAAAAGCATTGTTAACAGGATTATTTGTACAAGATAATGTGAATAAAGATGCCGTCACAGATAGATTGGCTTCAGTGGAGACTGATATGCCTCTAACATTGAGACAGCATAGAATAAGTGCGACATCATCATCGGAAGAGAGTAGTAACAAAGGTCAAAGACAGTTGACTGTATCG |
| miRNA intron | GTAAGTGTGCTCGCTTCGGCAGCACATATACTATGTTGAATGAGGCTTCAGTACTTTACAGAATCGTTGCCTGCACATCTTGGAAACACTTGCTGGGATTACTTCTTCAGGGACTTCTTAACCCAACAGAAGGCTCGAGAAGGTATATTGCTGTTGACAGTGAGCGCCGCTTGAAGTCTTTAATTAAATAGTGAAGCCACAGATGTATTTAATTAAAGACTTCAAGCGGTGCCTACTGCCTCGGACTTCAAGGGGCTAGAATTCAAGGGGCTACTTTAGGAGCAATTATCTTGTTTACTAAAACTGAATACCTTGCTATCTCTTTGATACATTTTTACAAAGCTGAATTAAAATGGTATAAATTAAATCACTTTTTTCAATTGTACTAACTTCGAGTCTTCTTTTTTTTTTTCACAG |
| VP16-AD, activation domain of GAD | TCGACGGCCCCCCCGACCGATGTCAGCCTGGGGGACGAGCTCCACTTAGACGGCGAGGACGTGGCGATGGCGCATGCCGACGCGCTAGACGATTTCGATCTGGACATGTTGGGGGACGGGGATTCCCCGGGTCCGGGA |
| Triplex (50bp RND) | CAAACAGGAATCGAATGCAACCGGCGCAGGAACACTGCCAGCGCATCAAC |
| FF4BS(Pv2), 9x for miRNABS | GCACCGCTTGAAGTCTTTAATTAAATAC |
| GAL4BS | GGAGTACTGTCCTCCG |
| Transferrin receptor, for hTFR-GFP | ATGGATCAAGCTAGATCAGCATTCTCTAACTTGTTTGGTGGAGAACCATTGTCATATACCCGGTTCAGCCTGGCTCGGCAAGTAGATGGCGATAACAGTCATGTGGAGATGAAACTTGCTGTAGATGAAGAAGAAAATGCTGACAATAACACAAAGGCCAATGTCACAAAACCAAAAAGGTGTAGTGGAAGTATCTGCTATGGGACTATTGCTGTGATCGTCTTTTTCTTGATTGGATTTATGATTGGCTACTTGGGCTATTGTAAAGGGGTAGAACCAAAAACTGAGTGTGAGAGACTGGCAGGAACCGAGTCTCCAGTGAGGGAGGAGCCAGGAGAGGACTTCCCTGCAGCACGTCGCTTATATTGGGATGACCTGAAGAGAAAGTTGTCGGAGAAACTGGACAGCACAGACTTCACCAGCACCATCAAGCTGCTGAATGAAAATTCATATGTCCCTCGTGAGGCTGGATCTCAAAAAGATGAAAATCTTGCGTTGTATGTTGAAAATCAATTTCGTGAATTTAAACTCAGCAAAGTCTGGCGTGATCAACATTTTGTTAAGATTCAGGTCAAAGACAGCGCTCAAAACTCGGTGATCATAGTTGATAAGAACGGTAGACTTGTTTACCTGGTGGAGAATCCTGGGGGTTATGTGGCGTATAGTAAGGCTGCAACAGTTACTGGTAAACTGGTCCATGCTAATTTTGGTACTAAAAAAGATTTTGAGGATTTATACACTCCTGTGAATGGATCTATAGTGATTGTCAGAGCAGGGAAAATCACCTTTGCAGAAAAGGTTGCAAATGCTGAAAGCTTAAATGCAATTGGTGTGTTGATATACATGGACCAGACTAAATTTCCCATTGTTAACGCAGAACTTTCATTCTTTGGACATGCTCATCTGGGGACAGGTGACCCTTACACACCTGGATTCCCTTCCTTCAATCACACTCAGTTTCCACCATCTCGGTCATCAGGATTGCCTAATATACCTGTCCAGACAATCTCCAGAGCTGCTGCAGAAAAGCTGTTTGGGAATATGGAAGGAGACTGTCCCTCTGACTGGAAAACAGACTCTACATGTAGGATGGTAACCTCAGAAAGCAAGAATGTGAAGCTCACTGTGAGCAATGTGCTGAAAGAGATAAAAATTCTTAACATCTTTGGAGTTATTAAAGGCTTTGTAGAACCAGATCACTATGTTGTAGTTGGGGCCCAGAGAGATGCATGGGGCCCTGGAGCTGCAAAATCCGGTGTAGGCACAGCTCTCCTATTGAAACTTGCCCAGATGTTCTCAGATATGGTCTTAAAAGATGGGTTTCAGCCCAGCAGAAGCATTATCTTTGCCAGTTGGAGTGCTGGAGACTTTGGATCGGTTGGTGCCACTGAATGGCTAGAGGGATACCTTTCGTCCCTGCATTTAAAGGCTTTCACTTATATTAATCTGGATAAAGCGGTTCTTGGTACCAGCAACTTCAAGGTTTCTGCCAGCCCACTGTTGTATACGCTTATTGAGAAAACAATGCAAAATGTGAAGCATCCGGTTACTGGGCAATTTCTATATCAGGACAGCAACTGGGCCAGCAAAGTTGAGAAACTCACTTTAGACAATGCTGCTTTCCCTTTCCTTGCATATTCTGGAATCCCAGCAGTTTCTTTCTGTTTTTGCGAGGACACAGATTATCCTTATTTGGGTACCACCATGGACACCTATAAGGAACTGATTGAGAGGATTCCTGAGTTGAACAAAGTGGCACGAGCAGCTGCAGAGGTCGCTGGTCAGTTCGTGATTAAACTAACCCATGATGTTGAATTGAACCTGGACTATGAGAGGTACAACAGCCAACTGCTTTCATTTGTGAGGGATCTGAACCAATACAGAGCAGACATAAAGGAAATGGGCCTGAGTTTACAGTGGCTGTATTCTGCTCGTGGAGACTTCTTCCGTGCTACTTCCAGACTAACAACAGATTTCGGGAATGCTGAGAAAACAGACAGATTTGTCATGAAGAAACTCAATGATCGTGTCATGAGAGTGGAGTATCACTTCCTCTCTCCCTACGTATCTCCAAAAGAGTCTCCTTTCCGACATGTCTTCTGGGGCTCCGGCTCTCACACGCTGCCAGCTTTACTGGAGAACTTGAAACTGCGTAAACAAAATAACGGTGCTTTTAATGAAACGCTGTTCAGAAACCAGTTGGCTCTAGCTACTTGGACTATTCAGGGAGCTGCAAATGCCCTCTCTGGTGACGTTTGGGACATTGACAATGAGTTT |
| GFP, for hTFR-GFP | ATGGTGAGCAAGGGCGAGGAGCTGTTCACCGGGGTGGTGCCCATCCTGGTCGAGCTGGACGGCGACGTAAACGGCCACAAGTTCAGCGTGTCCGGCGAGGGCGAGGGCGATGCCACCTACGGCAAGCTGACCCTGAAGTTCATCTGCACCACCGGCAAGCTGCCCGTGCCCTGGCCCACCCTCGTGACCACCCTGACCTACGGCGTGCAGTGCTTCAGCCGCTACCCCGACCACATGAAGCAGCACGACTTCTTCAAGTCCGCCATGCCCGAAGGCTACGTCCAGGAGCGCACCATCTTCTTCAAGGACGACGGCAACTACAAGACCCGCGCCGAGGTGAAGTTCGAGGGCGACACCCTGGTGAACCGCATCGAGCTGAAGGGCATCGACTTCAAGGAGGACGGCAACATCCTGGGGCACAAGCTGGAGTACAACTACAACAGCCACAACGTCTATATCATGGCCGACAAGCAGAAGAACGGCATCAAGGTGAACTTCAAGATCCGCCACAACATCGAGGACGGCAGCGTGCAGCTCGCCGACCACTACCAGCAGAACACCCCCATCGGCGACGGCCCCGTGCTGCTGCCCGACAACCACTACCTGAGCACCCAGTCCGCCCTGAGCAAAGACCCCAACGAGAAGCGCGATCACATGGTCCTGCTGGAGTTCGTGACCGCCGCCGGGATCACTCTCGGCATGGACGAGCTGTACAAGCGGACCGTC |
| SecTEV QSG | GGCGAGAGCCTCTTCAAGGGACCCAGGGACTACAACCCCATTAGCAGCACCATCTGCCACCTCACCCAGGAGTCCGACGGCCATACCACCAGCCTGTATGGAATCGGCTTTGGACCTTTCATCATCACCAACAAGCATCTGTTTCGGCGGAACAACGGCACCCTGCTGGTGCAGAGCCTGCATGGCGTGTTCAAGGTCAAGAACACAACTACCCTCCAGCAGCACCTGATCGATGGAAGAGACATGATCATCATCCGGATGCCAAAAGACTTTCCACCTTTTCCCCAAAAACTCAAATTCCGGGAGCCTCAGAGAGAAGAGCGGATCTGTCTGGTGACCACAAATTTCCAGACAAAAAGCATGAGCAGCATGGTGTCCGATACAAGCAGCACATTCCCATCCAGCGATGGCATCTTCTGGAAACATTGGATTCAGACAAAAGATGGCCAGTGCGGAAGCCCACTCGTCTCCACTAGAGACGGGTTCATCGTGGGGATTCACAGCGCTAGCAATTTTGGAAATACCAATAATTACTTTACATCCGTCCCAAAGAACTTCATGGAGCTGCTGACAAACCAGGAAGCACAGCAGTGGGTGAGCGGCTGGAGGCTGAACGCCGATTCCGTCCTGTGGGGCGGCCACAAGGTCTTCATGGTGAAGCCCGAAGAGCCTTTCCAGCCAGTGAAGGAGGCCACTCAACTGATGAAC |
